# Dihydroartemisinin inhibits prostate cancer via JARID2/miR-7/miR-34a-dependent downregulation of Axl

**DOI:** 10.1038/s41389-019-0122-6

**Published:** 2019-02-19

**Authors:** Juliano D. Paccez, Kristal Duncan, Durairaj Sekar, Ricardo G. Correa, Yihong Wang, Xuesong Gu, Manoj Bashin, Kelly Chibale, Towia A. Libermann, Luiz F. Zerbini

**Affiliations:** 1 International Centre for Genetic Engineering and Biotechnology (ICGEB), Cape Town, South Africa; 2 Department of Integrative Biomedical Sciences, University of Cape Town, Cape Town, South Africa; 3 Sanford Burnham Prebys Medical Discovery Institute, La Jolla, CA USA; 4 Department of Pathology and Laboratory Medicine, Warren Alpert School of Medicine, Brown University, Providence, RI USA; 5 BIDMC Genomics, Proteomics, Bioinformatics and Systems Biology Center, Beth Israel Deaconess Medical Center and Harvard Medical School, Boston, MA USA; 6 South African Medical Research Council Drug Discovery and Development Research Unit, Department of Chemistry and Institute of Infectious Disease and Molecular Medicine, University of Cape Town, Cape Town, South Africa

## Abstract

Axl expression is deregulated in several cancer types, predicts poor overall patient survival and is linked to resistance to drug therapy. Here, we evaluated a library of natural compounds for inhibitors of Axl and identified dihydroartemisinin, the active principle of the anti-malarial drug artemisinin, as an Axl-inhibitor in prostate cancer. Dihydroartemisinin blocks Axl expression leading to apoptosis, decrease in cell proliferation, migration, and tumor development of prostate cancer cells. Dihydroartemisinin treatment synergizes with docetaxel, a standard of care in metastatic prostate cancer increasing overall survival of mice with human xenografts. Dihydroartemisinin control of miR-34a and miR-7 expression leads to inhibition of Axl expression in a process at least partially dependent on regulation of chromatin via methylation of histone H3 lysine 27 residues by Jumonji, AT-rich interaction domain containing 2 (JARID2), and the enhancer of zeste homolog 2. Our discovery of a previously unidentified miR-34a/miR-7/JARID2 pathway controlling dihydroartemisinin effects on Axl expression and inhibition of cancer cell proliferation, migration, invasion, and tumor formation provides new molecular mechanistic insights into dihydroartemisinin anticancer effect on prostate cancer with potential therapeutic implications.

## Introduction

Prostate cancer (PCa), is the most frequent solid cancer in aging males, and the third leading cause of cancer death in the US^1^. The metastatic disease is the most important cause of increasing morbidity and mortality of PCa. The development of the metastasis stage of the disease involves multiple events, including the progression to hormone-independent status, which leaves physicians with very few treatment options. Although there are effective treatments of local PCa, such as radiation therapy, surgery, and androgen ablation therapy, only a few drugs have demonstrated some efficacy against hormone-refractory metastatic disease, such as docetaxel, abiraterone, and enzalutamide^2–4^. One major prerequisite to develop more effective targeted therapies is the identification of the most relevant cellular targets and enhancing understanding of the key pathophysiological pathways driving PCa progression. In this context, our group recently demonstrated that Axl is a relevant therapeutic target for metastatic castration-resistant PCa (mCRPCa)^5^.

The receptor tyrosine kinase Axl belongs to the TAM (Tyro-3, Axl, and Mer) family and possesses transforming potential when overexpressed^6,7^. Activation of Axl occurs subsequent to the binding of growth arrest-specific gene 6 (Gas6) which contains an N-terminal γ-carboxyl-glutamic acid domain, in a vitamin K-dependent event^8–11^. Axl expression has been associated with pathways closely related to progression and development of tumors and inhibition of apoptosis, such as the phosphatidylinositol 3-OH kinase (PI3K) pathway, MAP kinases, STAT, and NF-κB signal transduction pathway^5,12,13^. Furthermore, Axl plays a role in the epithelial-mesenchymal transition (EMT), which is an important feature for the initiation of metastasis^14–17^.

Axl is deregulated in cancers such as prostate, breast, lung, and oesophageal carcinomas^5,8,18–25^. Its expression predicts poor overall patient survival in breast and pancreatic cancer patients^26,27^ and is linked to increased resistance to therapy^28–32^, indicating that targeting Axl may represent a novel therapeutic approach for cancer treatment.

Here, we evaluated a library of natural compounds to identify and characterize specific Axl-inhibitors. We identified dihydroartemisinin (DHA), the active metabolite of artemisinin, which has been used as an anti-malarial drug, as a strong Axl-inhibitor. We demonstrated that DHA inhibits Axl expression, leading to decreased proliferation, migration, and invasion, induction of apoptosis of PCa cells and inhibition of tumor development in vivo. Moreover, DHA synergizes with docetaxel, a standard of care in mCRPC treatment, and increases the survival of mice with PCa xenografts. We provide strong evidence that DHA treatment effects on Axl expression are mediated by inhibition of microRNAs (miR-34a and miR-7) that regulate Axl expression. DHA regulation of miR-34a and miR-7 expression is dependent on JARID 2 and EZH2, components of the Polycomb Complex Repressor 2 (PRC2), a complex of proteins involved in proliferation, pluripotency, and maintenance of the developmental stage in adults, that acts through the regulation of the chromatin structure mainly by methylation of histone H3 lysine 27 residue (H3K27)^33,34^. In summary, we have characterized a novel mechanism of action for DHA as a specific Axl-inhibitor in PCa, providing insights into the signaling pathways underlying the anticancer effects of DHA in PCa cells.

## Results

### Screening of natural compounds and identification of dihydroartemisinin as an inhibitor of prostate cancer cell proliferation

We previously demonstrated the expression and pathophysiological function of Axl in a panel of PCa cells^5^. Here, we extended our analysis by investigating the expression of Axl in an additional panel of PCa cells. The castration-resistant PCa cells, DU145 and PC-3 lack androgen receptor (AR), PSA, and 5α-reductase^35,36^, while C4, C4-2 and C4-2B are castration-resistant LNCaP clones. We observed that Axl mRNA and protein levels are expressed in C4, C4-2 and C4-2B cells at higher levels than LNCaP cells, but lower than in DU145 and PC-3 cells. LNCaP cells express very low levels of Axl compared to DU145 and PC-3 cells (Fig. S1A and B).

**Fig. 1:**
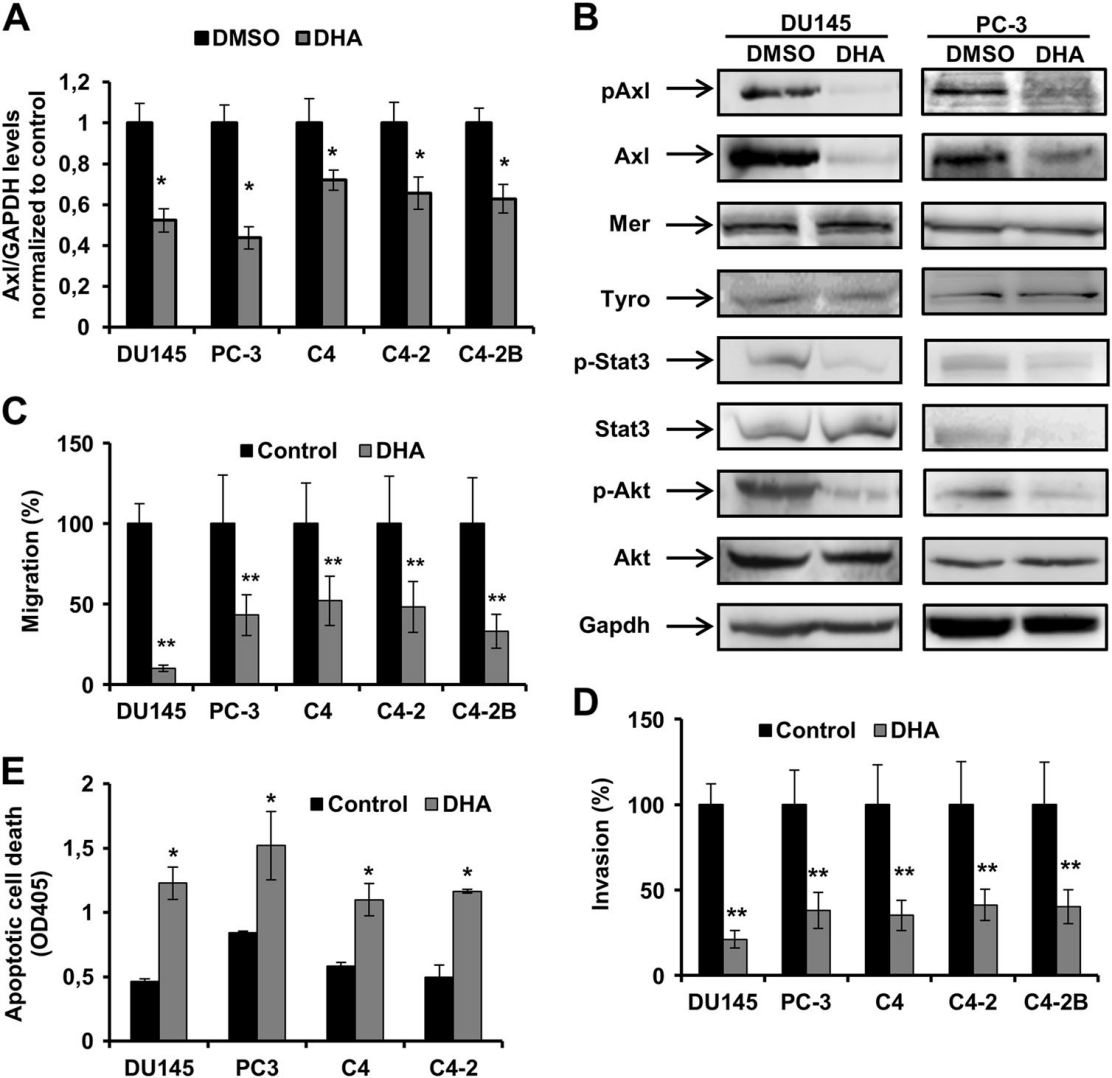
DHA treatment inhibits Axl expression and proteins involved in the Axl signaling pathway, reduces cell migration and invasion and induces apoptosis in mCRPCa cell lines. **a** qRT-PCR for Axl in PCa cells treated with 5 μM of DHA for 24 h. Values were normalized to Gapdh levels and to DMSO control. The experiment was performed in triplicate. Data are representative of 3 independent experiments; *p < 0.05 two-tailed Student’s *t*-test. **b** Immunoblot analysis of protein extracts obtained from DU145 and PC-3 treated with 5 μM DHA for 24 h using anti-phospho-Axl, anti-phospho-Akt and anti-phospho-Stat3 and anti-Axl, anti-Akt, anti-Stat3 and anti- GAPDH. **c** Migration and **d** invasion analysis of mCRPC cell lines 24 h post treatment with 5 μM of DHA. Cells were fixed and stained, and 3–5 random microscopic fields were counted. Data are representative of three independent experiments and all values shown are mean ± SD from a representative experiment; **p < 0.01, two-tailed Student’s *t*-test. **e** Apoptosis assay for mCRPCa cell lines treated with 5 μM of DHA for 8 h. DMSO 0.05% was used as a control. Data are representative of three independent experiments and all values shown are mean ± SD from a representative experiment; *p < 0.05, two-tailed Student’s *t*-test

We performed several cell-based assays utilizing a Natural Product Library (Selleck Chemicals) comprising of 144 natural compounds (Table S1), to identify inhibitors of PCa cell proliferation (Fig. S2). We analysed the solubility of the compounds in the media used to grow the panel of PCa cells and observed issues in 10 compounds (Table S2). The remaining 134 compounds were tested for inhibition of proliferation in PCa cells (DU145, PC-3, C4, C4-2, and C4-2B). Our analysis revealed a similar pattern of proliferation inhibition for PC-3, C4, C4-2, and C4-2B, but not DU145 (Fig. S3A). To select the most effective inhibitors of PCa cell proliferation, we ranked individual cells based on the inhibitory effects of the compounds (Fig. S3B). Amongst the top 10 most effective compounds, eight were common to PC-3, C4, C4-2, and C4-2B (Table S3). Only DHA demonstrated consistent inhibitory effects across all PCa cells tested. Consequently, we focused on the further evaluation of PCa proliferation inhibition by DHA. We previously demonstrated that Axl is a relevant therapeutic target for mCRPCa; Axl blockage inhibits the proliferation and migration of PCa cells as well as tumor formation in vivo^5^, we tested the hypothesis that DHA inhibits proliferation of mCRPCa cells via Axl modulation. We found that DHA reduced Axl mRNA expression in all PCa cell lines tested (Fig. 1a). Additionally, we analysed the impact of DHA treatment on the protein levels and phosphorylation status of Axl and other members of the TAM family (Mer and Tyro3) in different PCa cell lines (Fig. 1b). DHA leads to inhibition of both Axl protein expression and phosphorylation, without affecting the expression of Mer and Tyro3 proteins.

We, and others, have shown that Axl regulates the Akt/NF-κB pathway in cancer^5,12,24^. We evaluated the expression levels of components of the Akt/NF-κB signaling pathway by western blot analysis. As predicted, DHA reduces Akt and Stat3 protein phosphorylation, indicating Akt activation and inactivation of Stat3 signaling (Fig. 1b). Dose-response experiments to determine DHA concentration needed to inhibit PCa proliferation established the IC_50_ ranging from 3.9 to 5.1 µM in the panel tested (Table 1). Interestingly PCa cells expressing comparatively very low levels of Axl, such as LNCaP, exhibit higher IC_50_ when compared with cells expressing higher levels of Axl (DU145, PC-3, C4, C4-2, and C4-2B), suggesting that DHA acts in an Axl-dependent manner in PCa cell lines.

**Table 1:** Analysis of DHA effect in PCa cell lines

Cell line	IC_50_ ± SD (µM)	95% Confidence interval
**DU145**	4.8 ± 0.89	3.49–6.96
**PC-3**	5.1 ± 0.91	2.75–5.41
**C4**	3.9 ± 0.45	2.78–4.46
**C4-2**	4.9 ± 0.52	3.07–5.75
**C4-2B**	5.1 ± 0.55	3.59–5.86
**LNCaP**	22.1 ± 1.89	15.2–30.5

A dose-response curve was performed and inhibition of proliferation was analyzed. IC_50_ = 4.8 µM in DU145; 5.1 µM in PC-3; 3.9 µM in C4, 4.9 µM in C4-2, 5.1 μM in C4-2B and 22.1 μM in LNCaP

Other than proliferation, the Axl signaling pathway is important in inducing migration and invasion^5,8,14^. Transwell assays demonstrated that DHA treatment significantly inhibits migration and invasion of PCa cells (Fig. 1c, d). Moreover, the treatment of PCa cells for 4 h with DHA induces apoptosis (Fig. 1e). In contrast, DHA treatment of the non-cancer cells, CCD-18, PNT1A, and Cos-7, did not significantly reduce proliferation (Fig. S4A), indicating non-toxicity for non-cancer cells. Remarkably, analysis of Axl mRNA in this panel of non-cancer cell lines demonstrated lower levels of Axl transcript when compared to DU145 cells (Fig. S4B).

### DHA inhibition of prostate cancer cell proliferation and tumor formation in vivo is dependent on Axl expression

We observed that inhibition of the proliferation effect elicited by DHA is more prominent in PCa cell lines expressing higher levels of Axl. We hypothesized that DHA effects on PCa proliferation are Axl-dependent. We evaluated DHA effects on PCa cell lines upon knockdown of Axl expression by lentivirus encoding shRNA-Axl-gene^5^, compared to wild type cells. Lentivirus-shRNA-Axl as well as a lentivirus-shRNA-GFP (control) were used to infect PCa cells, resulting in ~90% reduction of Axl expression (Fig. S5)^5^.

**Fig. 2:**
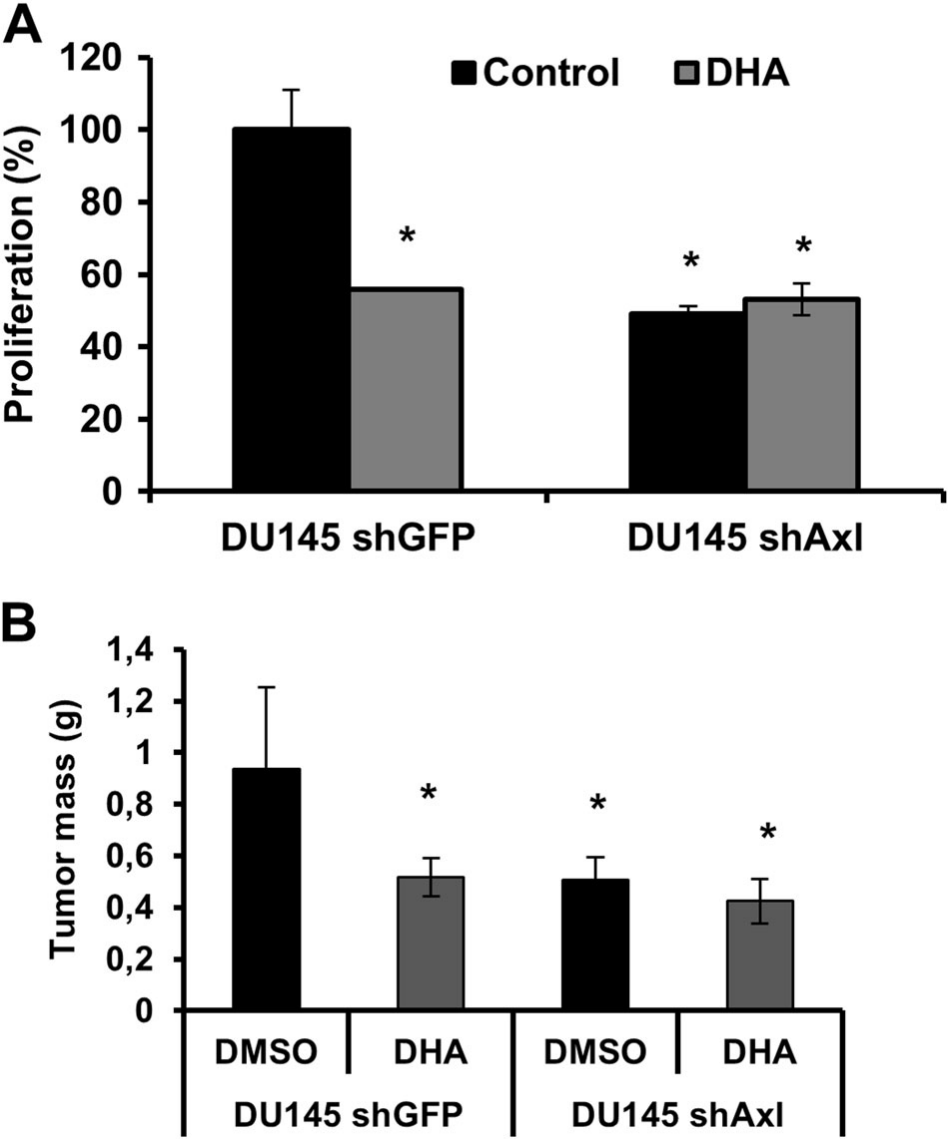
DHA inhibition of PCa proliferation and tumor formation in vivo is dependent on Axl expression. **a** Proliferation assay. DU145shGFP and DU145 shAxl cells were treated with DHA (5 μM) or DMSO (0.05%) for 24 h. Data were normalized to DU145shGFP treated with control and represented as percentage. Data shown are mean ± SD of triplicate independent experiments. Data are representative of 3 independent experiments. **b** DU145shGFP and DU145shAxl cells (5 × 10^6^) were implanted subcutaneously into the right flank of MF-1 mice. Mice were submitted to daily treatment with 40 mg/kg of DHA or DMSO (2 mL/kg). Tumor weight was measured 48 days after implantation. Values are represented as mean ± SD of six individuals; *p < 0.05, two-tailed Student’s *t*-test

DHA treatment inhibits DU145shGFP cell proliferation after 24 h incubation compared to cells treated with DMSO by 44% (*p* < 0.05), but has no effect on DU145shAxl (Fig. 2a). These findings confirm that DHA elicits its anti-proliferative effect at least partially in an Axl-dependent manner.

Previously, we demonstrated that Axl plays an important role in PCa tumor growth in vivo^5^. To determine whether DHA treatment impacts tumor formation in vivo, DU145 cells infected with LV-shRNA-GFP or LV-shRNA-Axl were subcutaneously implanted into the prostate of MF-1 nude mice. Mice were treated with DHA (40 mg/kg) or vehicle control (DMSO at 2 mL/kg) injected intraperitoneally for 50 days and examined for tumor formation and tumor weight. Treatment of mice implanted with the DU145shGFP cell line (control cells) with DHA significantly reduced tumor growth by 46% (Fig. 2b, *p* < 0.05) compared to DMSO vehicle-treated mice. Interestingly, treatment of mice implanted with the DU145shAxl cell line with DHA showed no significant difference in tumor burden when compared to DMSO vehicle-treated mice (Fig. 2b), reinforcing the notion that DHA acts in an Axl-dependent manner.

### DHA treatment enhances docetaxel efficacy in prostate cancer

Docetaxel is a semisynthetic taxane employed as a standard of care in mCRPCa treatment^2,37^. To test whether the treatment of docetaxel synergizes with DHA in reducing tumor growth, we evaluated the effect of DHA on docetaxel response in PCa cells. We defined the docetaxel IC_50_ in DU145 cells as 2 nM (Fig. S6). We evaluated the effect of docetaxel on the proliferation of PCa cells with siRNA-mediated knockdown of Axl (DU145shAxl) and showed that DU145shAxl cells are more sensitive to docetaxel treatment than DU145shGFP cells. Docetaxel IC_50_ in DU145shAxl cells is 0.6 nM, 3-fold lower in shAxl than in Axl+/+ (Fig. S6A). Isobologram analysis of PCa cells with combined docetaxel and DHA treatment using Calcusyn software revealed that the two drugs synergize (Fig. S6B). Pre-treatment of PCa cells for 24 h with DHA (2 µM) sensitized PCa cells to docetaxel and enhanced the growth-inhibitory effect of docetaxel (Fig. S6C). Cells pre-treated with DHA require 14-times lower concentrations of docetaxel compared to DMSO. These data reinforce the hypothesis that DHA treatment sensitizes PCa cells to and enhances docetaxel response in PCa. Furthermore, targeted Axl-inhibition could be an effective therapy for enhancing docetaxel efficacy in mCRPC.

### DHA synergizes with docetaxel to inhibit tumor formation in vivo

To determine whether DHA treatment has a synergistic effect on tumor formation in vivo when co-administrated with docetaxel, DU145 cells were implanted into MF-1 nude mice. Balanced cohorts with established mCRPC xenograft tumors were treated with vehicle DMSO (2 mL/kg) daily, DHA (40 mg/kg) daily, docetaxel (15 mg/kg) on days 1, 8, 15, 38, 45, 52 post inoculation, or DHA (40 mg/kg) daily combined with docetaxel (15 mg/kg) on days 1, 8, 15, 38, 45, 52 by intraperitoneal injection. 50 days later, mice were examined for tumor formation, tumor weight and IL-6 expression in the serum.

**Fig. 3:**
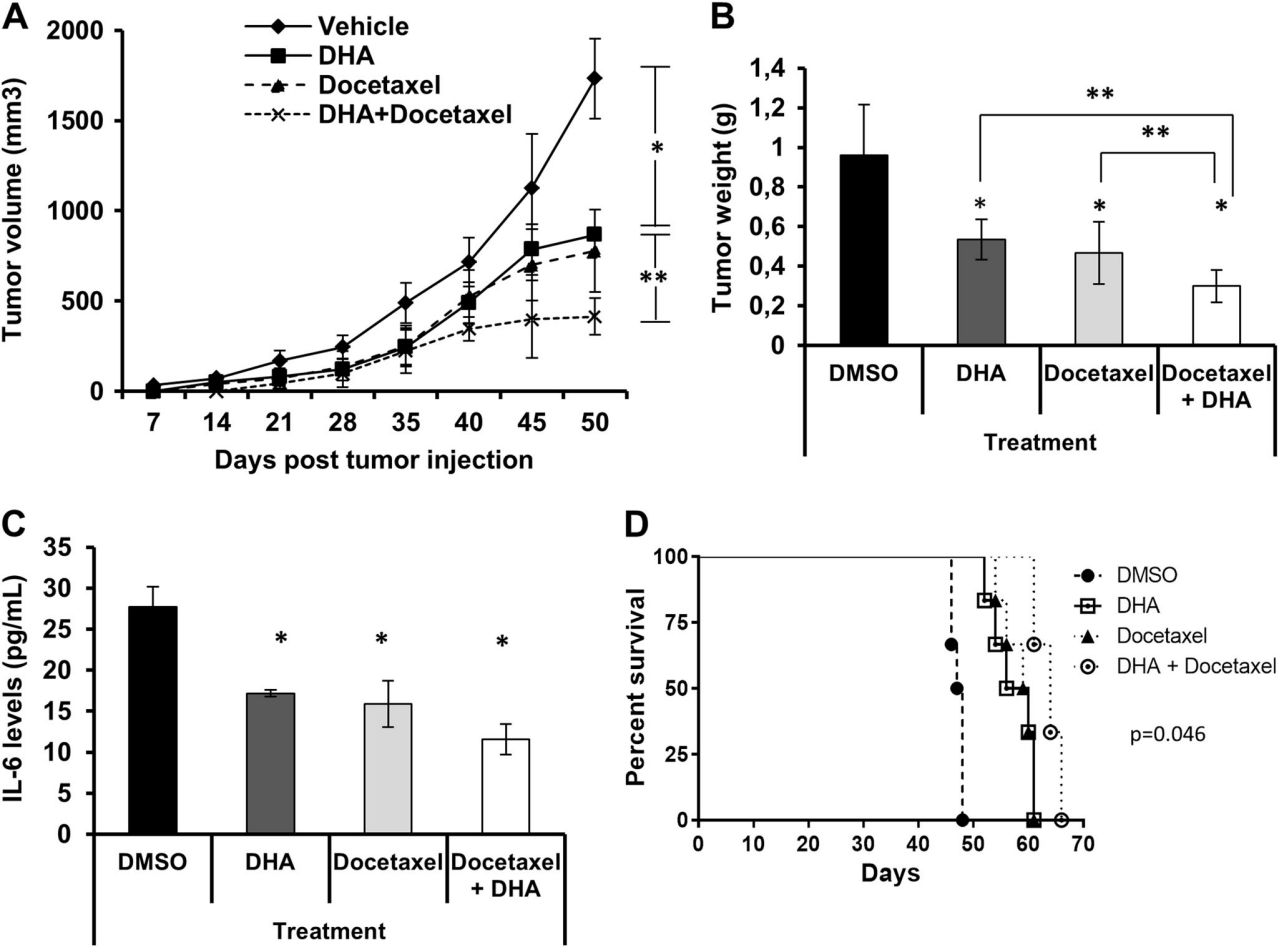
Synergistic effects of combinatorial therapy with DHA and docetaxel in vivo. DU145shGFP cells (5 × 10^6^) were implanted subcutaneously into the right flank of MF-1 mice. Mice were randomly divided into 4 groups (*n* = 6/group): Mice submitted to treatment with DMSO (2 mL/kg/daily), DHA (40 mg/kg/daily), docetaxel or combination of DHA (40 mg/kg/daily) and docetaxel (15 mg/kg/daily) as described in Material and methods. **a** Analysis of **a** tumor volume progression and **b** tumor volume. Values are represented as mean ± SD of six individuals; *p < 0.05 and **p < 0.001, two-tailed Student’s *t*-test. **c** Analysis of IL-6 levels in the blood of mice. Values are represented as mean ± SD of six individuals; * *p* < 0.05, two-tailed Student’s *t-*test. **d** Survival curve of mice treated DMSO (2 mL/kg/daily), DHA (40 mg/kg/daily), docetaxel (15 mg/kg/daily) or combination of DHA (2 mL/kg/daily) and docetaxel (15 mg/kg/daily)

DHA or docetaxel treatment resulted in a significant reduction of tumor growth and volume in mice when compared to those treated with DMSO. As observed in Fig. 3a, tumor growth is reduced by the use of docetaxel, DHA or a combination of both. At the end of the 50 days of the experiments, we observed reduction of 49.9% (*p* < 0.05) after treatment with DHA, 55.10% (*p* < 0.05) after treatment with docetaxel and 76.10% (*p* < 0.001) when combining both compounds. Analysis of dissected tumor at the end 50 days, demonstrated that tumor weight was reduced by 44.27% (*p* < 0.05) and 51.35% (*p* < 0.05) after treatment with DHA or docetaxel, respectively. Combination therapy with both compounds increased tumor weight reduction to 68.75% (*p* < 0.001) (Fig. 3b).

We evaluated the levels of IL-6 secreted in the blood of animals. IL-6 levels in blood predict poor outcomes in patients with localized tumors^38^. Previously, we demonstrated that Axl/Akt/NF-κB cascade via constitutive activation of NF-κB leads to IL-6 secretion in PCa^5^. We observed that treatment of mice with DHA, docetaxel or a combination of both, drastically reduced the levels of IL-6 secreted in mouse blood. The reduction in secreted levels of IL-6 in mice was 38.12% for DHA (*p* < 0.05), 42.77% for docetaxel (*p* < 0.05) and 58.41% for the combination of docetaxel and DHA (*p* < 0.05) compared to control (Fig. 3c). In an independent experiment utilizing the same cohorts, treatments, and conditions as described above, we evaluated the overall survival of mice submitted to the different treatment regimens. The experiment ended when tumors reached institutional limits (1500 mm^3^). Treatment with DHA and docetaxel prolonged overall survival compared with vehicle-treated controls. Treatment with DHA led to a median survival of 58 days compared to 48 days of the control group (Fig. 3d). This is similar to the survival seen for the docetaxel treated group (60 days). Moreover, the co-treatment of mice with DHA and docetaxel enhances survival to 64 days. Together, these results suggest that an Axl specific inhibitor may impact PCa treatment, leading to prolonged survival.

### Kinase screening and profiling indicates that DHA treatment does not affect Axl-kinase activity

We evaluated whether DHA treatment directly inhibits the Axl-kinase domain, or whether the decrease of Axl phosphorylation is directly linked to the decrease in Axl expression, as well as investigated the effect of DHA treatment on other tyrosine kinases. We performed a kinase screening and profiling assay, using the KINOME scanTK Kinase Assay Panel (DiscoverX).

It has three components: a kinase-tagged phage, a test compound, and an immobilized ligand that the compound competes with to displace the kinase. The amount of kinase bound to the immobilized ligand is determined using quantitative PCR of the DNA tag. The readout from assay is “percent of control”, where the control is DMSO and where a 100% result means no inhibition of the kinase by the test compound.

Under the conditions tested, DHA did not inhibit the kinase activity of any of the kinases screened, including Axl. (Fig. S7A). These results suggest that inhibition of Axl phosphorylation by DHA treatment is due to the inhibition of Axl protein expression. Furthermore, we investigated whether DHA-elicited inhibition of Axl protein expression is mediated by the proteasome pathway. DU145 cells were treated with DHA, MG132 (proteasome pathway inhibitor), and a combination of both. Four hour post-treatment, protein was extracted and Axl expression was analyzed by western blot. Treatment with MG132 did not affect the inhibition of Axl protein expression by DHA (Fig. S7B), indicating that DHA inhibition of Axl expression is not mediated by the proteasome pathway.

### DHA inhibits Axl expression by inducing miR-7 and miR-34a expression

**Fig. 4:**
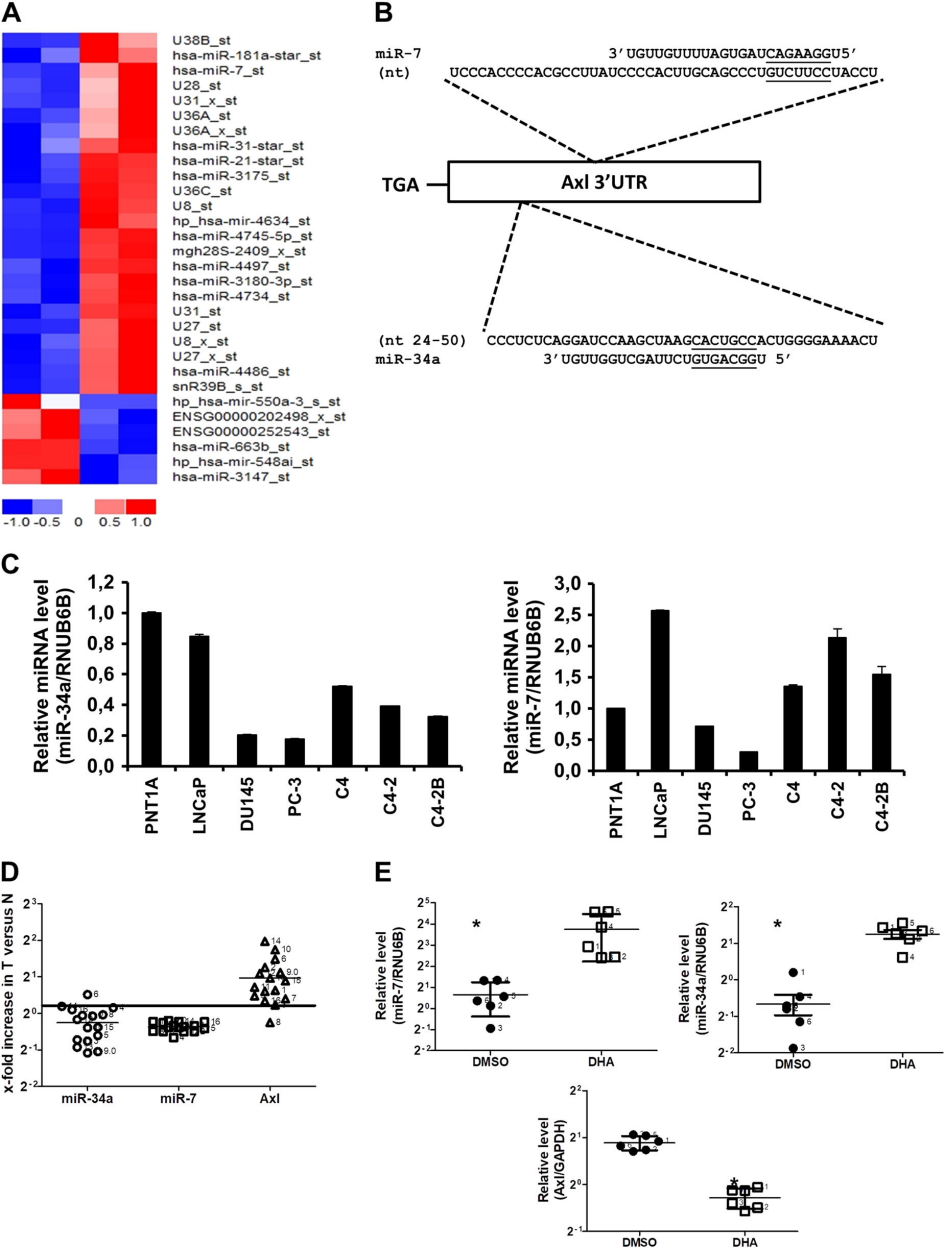
DHA regulates the expression of miRNAs in PCa cell lines. Cells were treated with DHA (5 μM) or DMSO (0.05%) for 24 h. **a** Heat map of miRNA microarray expression data. Cluster analysis classified the samples into groups based on miRNA expression levels in each sample. Red indicates high expression and green indicates relatively low expression of miRNA. **b** Schematic representation of the location of the putative miR-7 target site is shown in the Axl 3′-UTR. **c** RT-PCR analysis of miR-34 expression levels (left) and miR-7 (right) expression levels in PCa cell lines. ΔCt values graphed are relative to the endogenous control RNU6B small RNA and data were normalized to normal cell PNT1A. Data are representative of three independent experiments and all values shown are mean ± SEM from a representative experiment; *p < 0.05, two-tailed Student’s *t-*test. **d** RT-PCR analysis of expression levels of miR-34a, miR-7, and Axl in human PCa samples. Total RNA was collected from human tissue consisting of paired normal and tumor samples and was analyzed for miR-7, miR-34a, and Axl expression levels. ΔCt values graphed are relative to the endogenous control RNU6B small RNA (for miR-34a and miR-7) and GAPDH for Axl. Data are shown as the triplicate independent experiments; *p < 0.05, two-tailed, nonparametric Mann–Whitney test. **e** RT-PCR analysis of expression levels of miR-34a (right), miR-7 (left) and Axl (bottom) in tumor extracted from mice treated with 40 mg/kg of DHA or DMSO (2 mL/kg). Total RNA was collected from tumor and analyzed for miR-7 and miR-34a expression levels. ΔCt values graphed are relative to the endogenous control RNU6B small RNA. Data are representative of three independent experiments and all values shown are mean ± SD from a representative experiment; *p < 0.05, two-tailed, nonparametric Mann–Whitney test

Several reports demonstrated the involvement and deregulation of micro-RNAs in cancer^39–41^. Axl has been shown to be regulated by miR34a, miR199a, and miR199b^42^. To evaluate whether DHA treatment of PCa cells affects the expression of these miRNAs, we quantified their expression in PCa cell lines treated with DHA by qPCR. DHA treatment leads to a significant induction in miR-34a expression levels (2.18 fold), but not in miR-199a and miR-199b levels (Fig. S8A). To evaluate DHA treatment effects on other non-coding RNA, we performed microarray analysis using the GeneChip miRNA 3.0 Array (Affymetrix). Treatment of PCa cells with DHA led to differential expression of a distinct set of miRNAs (Fig. 4a). Validation of microarray data was performed by expression evaluation of some of the top regulated miRNAs by qRT-PCR. We confirmed that miR-7, miR-550, miR-548, miR-663, and miR-4634 exhibited the same trend of expression as observed in the microarray data (Fig. S8B). In order to identify non-coding RNAs that are likely to directly target Axl expression among this set of deregulated non-coding RNAs, we applied a bioinformatics approach to screen the 3′-UTR of the Axl transcript for complementary seed sequences of miRNAs. Using the *microRNA.org–Targets and Expression* database^43,44^ we identified miR-7 as an Axl repressor candidate (Fig. 4b).

We evaluated miR-7 and miR-34a expression levels in a panel of PCa cell lines. PCa cell lines expressing higher levels of Axl (DU145 and PC-3) express lower levels of miR-34a and miR-7, while PCa cells expressing lower levels of Axl (LNCaP, C4, C4-2, and C4-2B) express higher levels of these miRNAs (Fig. 4c). Likewise, we evaluated the relevance of miR-7 and miR-34a expression and its inverse correlation with Axl expression levels in clinical PCa samples. We analyzed 16 matched pairs of normal and PCa tumor patient samples. Characteristics of each sample including Gleason score, stage, and grade are described in Table S4. While Axl mRNA levels are consistently upregulated in tumor samples compared with matched normal tissue, miR-7 and miR-34a are downregulated (Fig. 4d) independent of tumor stage (Fig. S9). The observation that miR-7 and miR34a are inversely correlated to Axl levels in both PCa cell lines and clinical tissues, together with our finding that DHA treatment leads to inhibition of Axl and induction of miR-7 and miR-34a, persuaded us to determine whether treatment of mice with DHA affects levels of miR-7 and miR-34a in vivo. This analysis demonstrated significant increase in miR-7 and miR-34a levels, as well as reduction in the levels of Axl in tumors from mice treated with DHA (Fig. 4e).

**Fig. 5:**
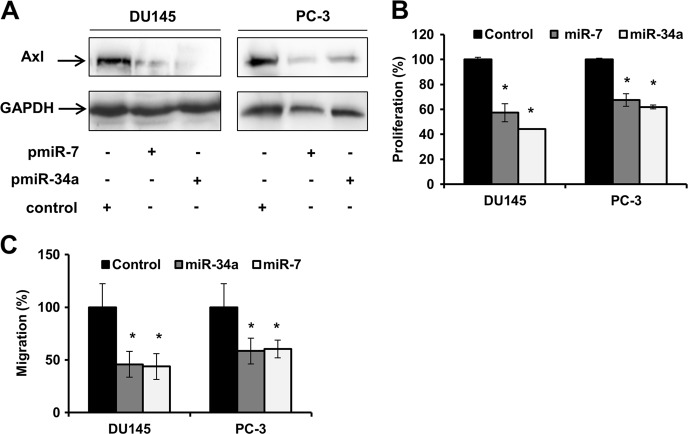
Expression of miR-7 and miR-34a inhibits Axl protein expression as well as proliferation and migration of in PCa cells. **a** Western blot analysis of Axl expression in DU145 and PC-3 cells transfected with miR-7 or miR-34a expression vectors and a vector control. **b** Proliferation assay of DU145 and PC-3 cells transfected with miR-7 or miR-34a expression vectors. Proliferation was measured 48 h post transfection. Data are representative of three independent experiments and all values shown are mean ± SD from a representative experiment; *p < 0.05, two-tailed Student’s *t*-test. **c** Migration assay of DU145 and PC-3 cells transfected with miR-7 or miR-34a expression vectors. Migration was measured 24 h post transfection. Migrating cells were fixed and stained, and three to five random microscopic fields were counted. Data are representative of three independent experiments and all values shown are mean ± SD from a representative experiment; *p < 0.05, two-tailed Student’s *t-*test

To gain further insights into Axl regulation by miR-7 and miR-34a, DU145 and PC-3 cells were transfected with miR-7 and miR-34a expression vectors as well as a control vector. Axl protein expression was analyzed in whole-cell extracts by western blot analysis. Expression of miR-7 or miR-34a results in the inhibition of Axl protein expression (Fig. 5a). Additionally, we evaluated the effect of the expression of miR-7 and miR-34a on proliferation and migration of PCa cells. As observed in Fig. 5b, both miR-7 and miR-34a reduce cell proliferation. The proliferation of PC-3 and DU145 cells are reduced by 32.2% (*p* < 0.05) and 42.3% (*p* < 0.05) after expression of miR-7. Likewise, the proliferation of PC-3 and DU145 cells are reduced by 38.8% (*p* < 0.05) and 56.3% (p < 0.05) after the expression of miR-34. Analysis of cell migration (Fig. 5c) demonstrated that the expression of miR-7 leads to inhibition of 39.7% (*p* < 0.05) in PC-3 and 56.3% (*p* < 0.05) in DU145. Migration of PC-3 and DU145 was similarly reduced by 41.6% (*p* < 0.05) and 54.2% (*p* < 0.05) after miR34a expression. These data provide strong evidence that miR-7 and miR-34a play critical roles in the DHA-mediated inhibition of Axl expression.

### DHA regulation of miR-7 and miR-34 and inhibition of Axl expression is mediated by polycomb repress complex 2 system

To gain insight into the functional and biological pathways that are involved in the DHA-mediated regulation of miRNAs and inhibition of Axl expression, we performed transcriptional profiling of DU145 cells treated with DHA (5 µM) or DMSO (0.05%) for 6 h. Transcriptional profiling analysis revealed 422 genes as differentially expressed in DHA-treated DU145 cells (Excel Spreadsheet 1 in Supplementary Information). Systems biology analysis of the genes differentially expressed upon DHA treatment indicates that DHA treatment interferes with a set of genes involved in proliferation and cell growth as well as cell and tissue development functions (Fig. S10). Our analysis indicates inhibition of JARID2 (Jumonji, AT-rich interaction domain containing 2) expression (data not shown), a DNA-binding component of the polycomb repress complex proteins (PRC2)^45,46^. PRC2 is one of the two classes of the polycomb group (PcG) proteins that are required for establishing and maintaining cellular memory and are involved in cellular processes such as proliferation and pluripotency^33^. PcG proteins form several complexes that function primarily through the regulation of chromatin structure^34,47^. PRC2 is a methyltransferase complex that acts specifically on histone H3 lysine 27 (H3K27) and is composed of four core components: enhancer of zeste homolog 2 (EZH2), Eed, Suz12, and JARID2^45,46,48^. EZH2 is the catalytic component of PRC2 and functions as a histone methyltransferase for H3K27 residues which is associated with repressed chromatin states and is widely distributed among genes encoding developmental regulators^49–51^. JARID2 interacts with EZH2 in the chromatin, stimulating its histone H3K27 methyltransferase activity^52^. In the absence of JARID2, PRC2 is recruited late to its target genes and its enzymatic function is diminished^52^.

**Fig. 6:**
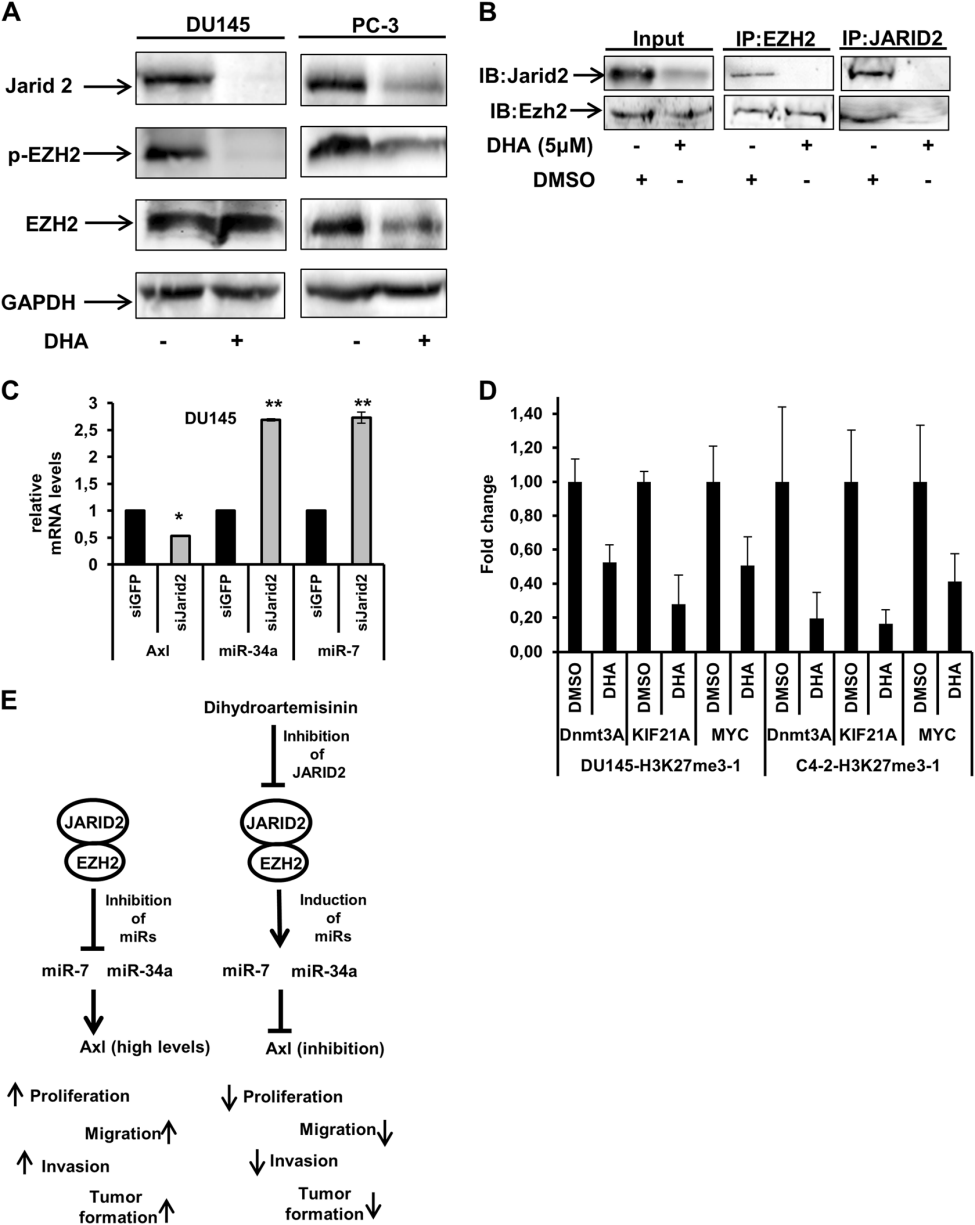
DHA inhibits proteins involved in the polycomb repressive complex. **a** Immunoblot analysis of protein extracts obtained from DU145 and PC-3 treated with DHA (5 μM) using anti-phospho EZH2, and anti-JARID2, anti-EZH2 and anti-GAPDH. **b** DHA Treatment of PCa cells inhibits JARID2-EZH2 interaction. DU145 cells were treated with 5 µM of DHA and DMSO (0.05%) and protein extracts were immunoprecipitated using anti-JARID2 or anti-EZH2 antibodies. The interaction between the proteins was detected by anti-EZH2 (right) or anti-JARID2 antibodies. **c** Inhibition of JARID2 protein regulates miR-34a and miR-7 expression and Axl expression. RT-PCR analysis of miR-34a, miR-7, and Axl expression levels of in DU145 cells lacking JARID2 expression. DU145 were transfected with JARID2 siRNA or GFP siRNA duplexes (50 nM). Total RNA was collected and was analyzed 24 h post transfection. ΔCt values graphed are relative to the endogenous control RNU6B small RNA (for miR-34a and miR-7) and GAPDH for Axl. Data are representative of three independent experiments and all values shown are mean ± SD from a representative experiment; *p < 0.05, two-tailed Student’s *t-*test. **d** ChIP-qPCR analysis demonstrating the effect of DHA treatment on DU145 and C4-2 cells on H3K27me3 at specific gene loci. PCa cell lines were treated with 5 uM of DHA or DMSO and subjected to ChIP analysis using antibodies against H3K27me3 or control IgG. The signals of H3K27me3 at the indicated genomic locations were normalized to that obtained from IgG ChIP at the same location to calculate their enriched signal. **e** Schematic representation of DHA inhibition of Axl expression via regulation of microRNAs and proteins of the polycomb repressive complex 2

We performed western blot analysis of JARID2 and EZH2 protein expression in DU145 and PC-3 cells treated with 5 µM of DHA for 24 h. DHA treatment resulted in the inhibition of JARID2 expression and reduced levels of p-EZH2 expression, the phosphorylated form of EZH2 (Fig. 6a). Furthermore, we investigated whether DHA treatment interferes with JARID2-EZH2 interaction, Immunoprecipitation of JARID2 or EZH2 proteins followed by detection with anti-EZH2 or anti-JARID2 antibodies in cells treated with DHA (5 µM) or DMSO (0.05%). Control demonstrates that JARID2 or EZH2 are not co-immunoprecipitated in cells treated with DHA, thus inhibition of JARID2 by DHA disrupts the formation of this complex (Fig. 6b).

We knocked down JARID2 in DU145 using siRNA (Fig. S10A) and evaluated miR-7, miR-34a, and Axl expression levels. Cells lacking JARID2 expression exhibit higher levels of miR-7 (2.72 fold) and miR-34a (2.68 fold) and lower levels of Axl (0.533 fold) (Fig. 6c). We did not observe any significant differences in miR-7 and miR-34a levels when JARID2 knockdown cells are treated with DHA (Fig. S10B and C), indicating that DHA modulation of miR-7 and miR-34a levels is JARID2 dependent.

Considering the above, we investigated the effect of DHA-mediated inhibition of JARID2 and JARID2-EZH2 interaction on EZH2 H3K27 methyltransferase activity. We performed Chromatin Immunoprecipitation (ChIP) analysis using H3K27me3 antibodies, followed by qPCR from the immunoprecipitated DNA of DU145 and C4-2 cells treated with DHA or DMSO. PCa cells treated with DHA presented decreased levels of H3K27me3 at specific gene loci (ANKRD30BL, Dnmt3A, KIF21A and MYC promoter) compared with cells treated with DMSO (Fig. 6d and Fig. S11), suggesting that DHA affects H3K27me3 methyltransferase activity.

## Discussion

Identifying relevant cellular targets and patient populations is necessary for developing and testing targeted therapies. Previously, we identified the tyrosine kinase receptor Axl as a potential target for PCa therapy^5^. Increased Axl levels have been linked to resistance to Imatinib in gastrointestinal stromal tumors, Lapatinib in HER-2 positive breast tumor cells^53^, BMS-754087 in Rhabdomyosarcoma^54^, and metformin in PCa^55^, as well as erlotinib in non-small cell lung cancer (NSCLC)^32^. Moreover, Axl overexpression has been described as mediating resistance to PI3K inhibitors^30^.

Numerous studies have described the development of Axl-inhibitors^26,56,57^. The development of small molecules such as R428 (also known as BGB324)^26^ and monoclonal antibodies such as YW327.6S2^58^ are promising. However, the development of specific Axl-inhibitors represents a major challenge due to the lack of an Axl structure and the fact that it shares high structural similarity with the rest of the TAM family and other kinases of the human kinome^57^. Therefore, screening of biologically and/or pharmacologically active natural compounds is a viable alternative approach, as several natural products and their semisynthetic derivatives are used in cancer chemotherapy.

In this study, the screening of a natural compound library has identified dihydroartemisinin (DHA) as a potent Axl-inhibitor in PCA cells. Our findings demonstrate that DHA inhibits proliferation and migration of PCa cells and tumor formation in vivo in an Axl-dependent manner. It leads to reduction of IL-6 levels, a mediator of morbidity and mortality in patients with mCRPCa^38^. Moreover, our group has shown that DHA regulates NF-κB and Akt in PCa which is in line with the previous reports^59^.

Co-treatment with DHA and docetaxel demonstrated synergistic effects against PCa both in vitro and in vivo. Docetaxel improves survival in patients with metastatic PCa. However, side-effects are observed in patients treated with this drug and patients become resistant to docetaxel^37^. Side-effects to docetaxel treatment may be minimized if combined with DHA, since DHA treatment can decrease docetaxel dosage needed for efficacy, leading to improved quality of life during treatment. As Axl plays a role in resistance to various cancer drugs, and DHA reduces Axl expression in PCa, combination of docetaxel with DHA may also delay or prevent resistance to docetaxel.

Besides some evidence of the anticancer potential of artemisinin and its derivatives, particularly DHA, for different types of cancers, there is little information about its mechanism of action, thus our findings are novel. We demonstrated that DHA does not inhibit Axl-kinase itself or via proteasome degradation. In fact, DHA-induced inhibition of Axl expression appears to be mediated by regulating specific micro RNAs. DHA treatment leads to increased expression of miR-34a and miR-7. MiR-34a has been shown to be a tumor suppressor in different types of cancers such as glioma, breast and PCa, and a part of the p53 tumor suppressor network, and regulates Axl expression^42,60^. MiR-7 targets and downregulates oncogenic factors in cancer-associated signaling pathways including EGF, Bcl-2, Raf1, and Akt/PI3K^61^. Using bioinformatics analysis, we identified miR-7 as having complementary seed sequences to the 3′-UTR of the Axl-gene. Ectopic expression of miR-7 and miR-34a inhibits Axl expression. miR-7 and miR-34a expression levels are inversely correlated with Axl expression in clinical PCa samples. Tumor samples derived from xenograft mice treated with DHA exhibit higher levels of miR-7 and miR-34a expression, suggesting that these miRNAs play a critical role in PCa.

Further gene expression and bioinformatics analysis in PCa cells treated with DHA indicates that DHA inhibits the expression of JARID2, a DNA-binding protein which is a component of the polycomb repressive complex 2 (PRC2)^46^. PRC2 regulates gene silencing through chromatin reorganization and histone methylation^45,48^. EZH2, another component of the PRC2 complex, has been described as an oncogene which is deregulated in prostate and breast cancer, and implicated in cancer progression and poor prognosis^62,63^. EZH2 catalyses trimethylation of H3K27, creating repressive chromatin structures over long genomic distances^64^. In fact, H3K27me3 is a signature of PRC2 activity and is involved in gene silencing through several mechanisms^65^. Treatment of PCa cells with DHA decreases the enrichment of trimethylation of H3K27 in specific gene loci, reinforcing the notion that DHA inhibits the formation of the PRC2 complex.

Regulation of miRNAs by PRC2 proteins is pivotal in cancer^66^. Nevertheless, a recent report has described a positive regulation of Axl by EZH2, independent of histone or DNA methylation in glioblastoma^67^. Induction of miR-7 and miR-34a in cells treated with DHA or cells not expressing JARID2 may indicate a role of PRC2 in the regulation of these miRNAs. In fact, we have demonstrated that inhibition of JARID2 by DHA treatment causes inhibition of EZH2, avoiding the establishment of PRC2 and inducing miR-7 and miR-34a, which in turn inhibits Axl (Fig. 6e). Even though we demonstrated a direct link between inhibition of PRC2 complex components i.e., JARID2 by DHA, it is important to note that the exact mechanism by which DHA inhibits JARID2, which may include direct interaction, remains unclear and is under investigation by our group. In conclusion, we provide strong evidence that DHA inhibits Axl expression in PCa via regulation of microRNAs and proteins of the polycomb repressive complex 2.

## Materials and methods

### Cell lines and culture

Human PCa cell lines C4, C4-2, and C4-2B were kindly provided by Dr. Steven Balk (Beth Israel Deaconess Medical Center and Harvard University); DU145, PC-3, and LNCaP, as well as non-cancer cell lines CCD-18 and Cos-7, were purchased from ATCC. PNT1A, a normal prostate epithelial cell line, was purchased from Sigma Aldrich. Knockdown cell lines DU145shGFP and DU145shAxl cells were previously described^5^. Details from authentication and culture methods are described in Supplementary data.

### Plasmids and transfection assays

Transfection was performed as previously described^5,68,69^ using Lipofectamine plus reagent (Invitrogen) and plasmids, allowing expression of microRNA precursors; pCMV-miR-7 (Origene, SC400648), pCMV-miR-34a (Origene, SC400356) and pCMV as a control.

### Reagents

Library of Natural Products (Cat No.L1400Selleck Chemicals). Dihydroartemisinin (D7439), docetaxel (01885) from Sigma-Aldrich, MG132 (474791), Calbiochem San Diego, CA, USA) were dissolved in DMSO (Sigma-Aldrich). siRNA-JARID2 (sc-60872, Santa Cruz Biotechnology) was used following the manufacturer’s recommendation.

### Isobologram

Analysis of docetaxel and DHA combinatorial treatment was demonstrated by isobologram, and calculated using Calcusyn software (Biosoft) as previously described^70^.

### Immunoprecipitation and western blot

Immunoprecipitation and Western blot analyses were performed as previously described^5,68,69^ using the primary antibodies Mer (#9178), Tyro3 (#5585), Axl (#4977), p-Akt (#92765), Akt (#9272), p-Stat3 (#9132), Stat3 (#9131), EZH2 (#4905), p-EZH2 (#27888) and GAPDH (Cell Signalling Technology, Beverly, MA, USA), JARID2 (sc-134548 - Santa Cruz Biotechnology) and p-Axl (AF2228) (R&D Systems, Minneapolis, MN, USA). For immunoprecipitation assays, 500 µg of total protein were immunoprecipitated using anti-JARID2 or anti-EZH2 antibodies coupled to protein-G agarose.

### Proliferation and apoptosis assays

Proliferation and apoptosis assays were performed using Cell Proliferation Kit I (MTT; Roche, Basel, Switzerland) and Apoptotic Cell Death Detection ELISA (Roche), respectively, according to the manufacturer’s protocol.

### Drug treatment

Cells were treated with compounds in their particular medium for 24 h (proliferation analysis) or 6 h (apoptosis analysis). For determination of IC_50_, cells were incubated with different concentrations of DHA (0.0048–5 µM) and Docetaxel (2.4–625 nM of docetaxel) for 24 h. Proliferation was analyzed. For combinatorial effect analysis, PCa cells were pre-treated with DHA (2 µM) for 24 h and treated with different concentrations (2.4–625 nM) of docetaxel. Proliferation was measured 24 h after docetaxel treatment.

### Invasion and migration assays

Cell migration and invasion assays were performed as previously described^38^, using a modified transwell chamber migration assay and invasion assay matrigel-coated membrane (BD Biosciences, Bedford, MA, USA).

### Real-time PCR

Total RNA was harvested using QIAshredder (Qiagen, Valencia, CA, USA) and the RNeasy mini kit (Qiagen) from tissue samples or cells, as recommended by the manufacturer. Primers used for real-time PCR are described in Supplementary information, and conditions were as described previously^5^.

### Quantification of miRNA by RT-PCR

Quantification of miRNAs was performed using specific and sensitive quantitative RT-PCR^71^ on LightCycler®480 InstrumentII (Roche). Primers and adaptors sequence are described in Supplementary information.

### Animal experiments

Eight-week-old male MF-1 nude mice, obtained from the University of Cape Town, were bred at the University’s animal facility and housed in a pathogen-free environment. Details of procedures, groups, and treatment schedule are described in details in Supplementary information. All procedures with animals were reviewed and approved by the Animal Research Ethics Committee of the University of Cape Town.

### IL-6 detection

IL-6 was assayed as described^5,38^ using ELISA (InvitrogenKHC0061C, Carlsbad, CA, USA) according to the manufacturer’s protocol.

### Tissue samples

Histopathological-confirmed PCa biopsies and the corresponding adjacent normal tissue samples were obtained from the Rhode Island Hospital, Brown University, Providence, Rhode Island, USA. Informed consent was obtained from all participants, while ethical approval for this study was obtained from the Ethics Committee of the Brown University (approved protocol number 015/005). Samples were stored in RNA later solution (Qiagen) at −80 °C until nucleic acid extraction was performed following the manufacturer’s instructions.

### Kinase screening and profiling

Kinase screening and profiling were performed using the *scan*TK^℠^ Kinase Assay Panel (DiscoverX). We used 7 μM of DHA and the experiment was performed according to the manufacturer’s instructions.

### Microarrays analysis

RNA samples for microarray analysis were obtained using QIAshredder (Qiagen) and RNeasy Mini Kit (Qiagen) and converted into cRNA following the manufacturer’s instructions (Affymetrix, Santa Clara, CA, USA). Details of analyses are described in Supplementary material.

### Microarray data deposition

Dataset have been deposited in the Gene Expression Omnibus under GSE122625

### Chromatin immunoprecipitation

Chromatin immunoprecipitation was performed as previously described^72^. Details are described in Supplementary Information.

### Statistical analysis

Unpaired Student *t*-test was used for comparison of drug treatment in PCa cell lines. Gehan-Breslow-Wilcoxon test was used for analysis of the survival curve. All were performed using GraphPad Prism 5.00 (GraphPad Software).

## Supplementary information

Supplementary Information.
