# Supplementary material for: Dihydroartemisinin inhibits prostate cancer via JARID2/miR-7/miR-34a-dependent downregulation of Axl
Source: Oncogenesis. 2019 Feb 19;8(3):14. doi: 10.1038/s41389-019-0122-6 (PMC6381097; doi:10.1038/s41389-019-0122-6)
Supplement: Supplementary file 1 — Supplementary Information. [file 41389_2019_122_MOESM1_ESM.docx]

**Supplementary Material**

**Supplementary Material and methods**

**Cell lines authentication and culture**

Cells were authenticated by the cell bank using DNA profile (STR) and cytogenetic analysis. Cells were cultured in the appropriate media (Life Technologies, Carlsbad, CA) as described below and supplemented with 10% fetal bovine serum (FBS), 50U of penicillin/ml, and 50mg streptomycin/ml (Life Technologies, Carlsbad, CA) and maintained in a 5% CO2 humidified incubator at 37ºC. Growth media were DMEM for DU145, Cos-7 and CCD-18; Ham’s F12 for PC-3 and RPMI for C4, C4-2, C4-2B, LNCaP and PNT1A. Cell lines were routinely frozen within 2 passages after receipt from the cell bank and used within less than 6 months after recovery.

**Animal experiments**

Eight-week-old male MF-1 nude mice, obtained from the University of Cape Town, were bred at the University’s animal facility and housed in a pathogen-free environment. Animals were randomly divided into groups of 6 mice and subcutaneously implanted with PCa cell lines (5x10^6^/mice). Sample size calculation for equal variance t-test showed the sample size needed was six per group to achieve the statistical power of 0.8. Immediately before implantation, cell lines were trypsinized and re-suspended in DMEM with 10% fetal bovine serum. Cell viability was determined by trypan blue exclusion and a single cell suspension with 90% viability was used for implantation.

Evaluation of the DHA effect was performed in DU145shGFP and DU145shAxl cells. Each group was submitted to treatment with DHA (40mg/kg) injected intraperitoneally on a daily basis and tumor size (volume) was measured on a weekly basis after implantation. At the end of the experiment (50 days), animals were euthanized, tumors were carefully dissected and weighed, and lymph nodes were analyzed to determine metastases.

The analysis of co-treatment with DHA and docetaxel was performed in DU145shGFP. Each group was submitted to the following treatment with intraperitoneal injection: DMSO (2mL/kg) daily, DHA daily (40 mg/kg), docetaxel (15 mg/kg) on days 1, 8, 15, 38, 45, 52 post inoculation, or DHA (40 mg/kg) daily and docetaxel (15 mg/kg) on days 1, 8, 15, 38, 45, 52. Tumor size (volume) was measured on a weekly basis after implantation and the experiment was concluded when the average tumor reached 1500mm^3^. At the end of the experiment phlebotomy was performed by accessing the retrorbital venous plexus to obtain 150μL of blood from each mouse in order to measure serum IL-6 level. Animals were euthanized, tumors were carefully dissected and weighted. All procedures with animals were reviewed and approved by the Animal Research Ethics Committee of the University of Cape Town.

**Microarrays analysis**

RNA samples for microarray analysis were obtained using QIAshredder (Qiagen) and RNeasy Mini Kit (Qiagen) and converted into cRNA following the manufacturer’s instructions (Affymetrix, Santa Clara, CA, USA). Analyses were performed in two groups of experiments. In the first group, total RNA was extracted from tumors isolated from MF-1 mice submitted to treatment with DHA or control as described before. cRNAs were hybridized to the HT U133AAofAv2 (Affymetrix) array plate. In the second set of experiments, DU145 cells were treated with DHA (5μM) or DMSO (0.05%) for 6h. cRNAs were hybridized to GeneChip miRNA 3.0 Array (Affymetrix, #902017). Treatments were performed in duplicates. Array washing and staining were performed on the GeneChip GCAS Array as previously described [1]. Arrays were scanned on the GeneChip HT scanner (Affymetrix), image output files were examined for major chip defects and hybridization artifacts and then analyzed with Affymetrix GeneChip Microarray Analysis Suite 5.0 (MAS5) software (Affymetrix). High-quality arrays were analyzed using the Probe Logarithmic Intensity Error (PLIER) algorithm. The genes with the 90% lower confidence bound (LCB) of the fold change (FC) above 1.2 in a given group were considered to be differentially expressed[2].

**Chromatin immunoprecipitation**

Cells were cross-linked for 10 minutes with 1% formaldehyde. Cross-linking was terminated by the addition of 1/10 volume 1.25M glycine for 5min at room temperature followed by cell lysis and sonication, resulting in an average chromatin fragment size of 200bp. DNA was immunopreciptated by incubation with anti-Tri-Methyl-Histone H3 (Lys27) antibody (Cell signalling #9733) at 4°C adsorbed in Magnetic beads (Invitrogen 10003D) followed by wash and reversal of cross-linking.

Validation of ChIP was performed by real-time PCR for known targets of Tri-Methyl-Histone H3 (Lys27). We analyzed the amount of ANKRD30BL, Dnmt3A and KIF21A gene loci as well as MYC promoter DNAs bound to Tri-Methyl-Histone H3 (Lys27). We used real time quantitative PCR (SYBR green) to amplify the DNA fragment in the antibody precipitated DNA and the un-precipitated input DNA to calculate ΔC_T_ values. The R_Q_ values (R_Q_=2^−ΔCT^) reflecting the precipitated DNA as a percentage of the input DNA were normalized using the values found in the control (DMSO treated) for each cell line. Results are represented as mean±STD for replicate samples. Data are representative of at least three experiments.

**RT-PCR**

Real Time PCR was performed as described [3] and the sequences of the primers are as follows:

**Axl**

sense: 5’-TGTTTGGTGTTTCTGGGACA-3’

antisense: 5’-TCGCAGGAGAAAGAGGATGT-3’

**hGADPH**

sense: 5’-CAAAGTTGTCATGGATGACC-3’

antisense: 5’-CCATGGAGAAGGCTGGGG-3’

ANKRD30BL-K27me3-F: CTCGACCTCCCGAAATCATA

ANKRD30BL-K27me3-R: GAAGTGCATGCTTAGGTGCTT

Dnmt3A-K27me3-F: GTGGTGAAAGGATGCTGGAA

Dnmt3A-K27me3-R: CCAGCCCTCTTTCCTTTCTC

KIF21A-K27me3-F: TTGGGTAGGAAAGCTTTTGA

KIF21A-K27me3-R: TCTCTAGTTTTCCTTGTGGACAG

PCBD2-K27me3-F: TTGAGGTTGTGGATGATGGA

PCBD2-K27me3-R: CCAACCACACCTAGCATTCC

Myc_pro_F: AGGGCTTCTCAGAGGCTTG

Myc_pro_R: CGGCTCTTCCACCCTAGC​

Quantification of miRNAs was performed as previously described [4] and the sequences of adaptors and primers are as follows:

**miR-34a**

adaptor: 5’-CAGGTCCAG(T)_15_ACA-3’

sense: AGTGGCAGTGTCTTAGCTGG

antisense: AGGGTCCAGTTTTTTTTTTTTTTTACA

**miR-199a**

adaptor: 5’-CAGGTCCAG(T)_15_AAG-3’

sense: 5’-CAGCCCAGTGTTCAGACTACCT-3’

antisense: 5’-AGGGTCCAGTTTTTTTTTTTTTTTGAA-3’

**miR-199b**

adaptor: 5’-CAGGTCCAG(T)_15_AAG-3’

sense: 5’- GCAGCCCAGTGTTTAGACTATCT-3’

antisense: 5’-AGGGTCCAGTTTTTTTTTTTTTTTGAA-3’

**miR-663b**

adaptor: 5’-CAGGTCCAG(T)_15_TCC-3’

sense: 5’-AGTATGGCCCGGCCGTGA-3’

antisense: 5’-AGGTCCATTTTTTTTTTTTTTTCCT-3’

**miR-3147**

adaptor: 5’-CAGGTCCAG(T)_15_ACT-3’

sense: 5’-ACTTGGGCAGTGAGGAGGGT-3’

antisense: 5’-AGGTCCATTTTTTTTTTTTTTTTCA-3’

**miR-21**

adaptor: 5’-CAGGTCCAG(T)_15_ACA-3’

sense: 5’-CAACACCAGTCGATGGGCT-3’

antisense: 5’-AGGTCCAGTTTTTTTTTTTTTTTTACA-3’

**miR-31**

adaptor: 5’-CAGGTCCAG(T)_15_ ATG-3’

sense: 5’-CGTGCTATGCCAACATATTGC-3’

antisense: 5’- AGGTCCAGTTTTTTTTTTTTTTTATG-3’

**miR-7**

adaptor: 5’-CAGGTCCAG(T)_15_ACA-3’

sense: 5’-GCGCAGTGGAAGACTAGTGATTT-3’

antisense: 5’-AGGTCCAGTTTTTTTTTTTTTTTACA-3’

**miR-181a**

adaptor : 5’-CAGGTCCAG(T)_15_GGA-3’

sense: 5’-ACCATCGACCGTTGATTG-3’

antisense: 5’- CGGTCCAGTTTTTTTTTTTTTTTGGA-3’

**miR-4745**

adaptor : 5’-CAGGTCCAG(T)_15_CGC-3’

sense: 5’-CCAGCTCCCGGGACGG-3’

antisense: 5’- GTCCAGTTTTTTTTTTTTTTTCGC -3’

**miR-3175**

adaptor: 5’-CAGGTCCAG(T)_15_ACG-3’

sense: 5’-AGGGGAGAGAACGCAGTGA-3’

antisense: 5’-AGGTCCAGTTTTTTTTTTTTTTTACG-3’

**miR-3180**

adaptor: 5’-CAGGTCCAG(T)_15_GGC-3’

sense: 5’-CAGCGGAGCTTCCGGAG-3’

antisense: 5’- AGTCCAGTTTTTTTTTTTTTTTGGC-3’

**miR-548a**

adaptor: 5’-CAGGTCCAG(T)_15_GGG-3’

sense: 5’-GCGAAAGGTAATTGCAGTTTTT-3’

antisense: 5’-AGGTCCAGTTTTTTTTTTTTTTTGG-3’

**miR-550a**

adaptor: 5’-CAGGTCCAG(T)_15_CTC-3’

sense: 5’-CGAGTGCCTGAGGGAGTAAG-3’

antisense: 5’-AGGTCCAGTTTTTTTTTTTTTTTCTC-3’

**miR-4497**

adaptor: 5’-CAGGTCCAG(T)_15_GCC-3’

sense: 5’-CTCCGGGACGGCTGG-3’

antisense: 5’- GTCCAGTTTTTTTTTTTTTTTGCC-3’

**References**

1. de Vasconcellos JF, Laranjeira ABA, Leal PC, Bhasin MK, Zenatti PP, Nunes RJ, et al. SB225002 Induces Cell Death and Cell Cycle Arrest in Acute Lymphoblastic Leukemia Cells through the Activation of GLIPR1. *PLoS One* 2015;**10**:e0134783.

2. Gu X, Zerbini LF, Otu HH, Bhasin M, Yang Q, Joseph MG, et al. Reduced PDEF expression increases invasion and expression of mesenchymal genes in prostate cancer cells. *Cancer Res.* 2007;**67**:4219–26.

3. Paccez JD, Vasques GJ, Correa RG, Vasconcellos JF, Duncan K, Gu X, et al. The receptor tyrosine kinase Axl is an essential regulator of prostate cancer proliferation and tumor growth and represents a new therapeutic target. *Oncogene* 2013;**32**:689–98.

4. Balcells I, Cirera S, Busk PK. Specific and sensitive quantitative RT-PCR of miRNAs with DNA primers. *BMC Biotechnol.* 2011;**11**:70.

**Supplementary Figures**

**
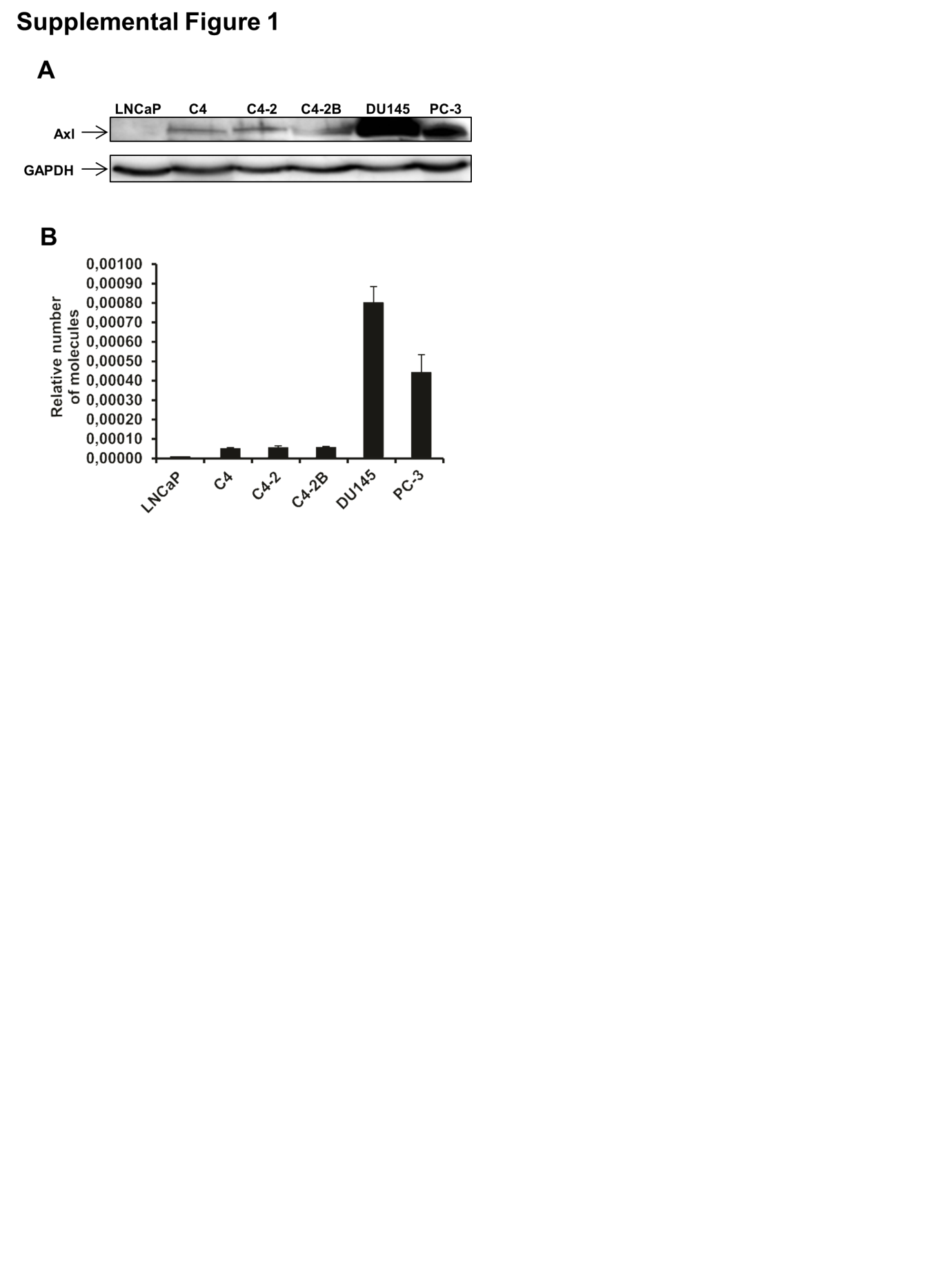
**

Figure S1. Axl basal levels in prostate cancer cell lines. (A) Western blot analysis of Axl basal levels in DU145, PC-3, C4, C4-2 and C4-2B cells (B) qRT-PCR for Axl mRNA of prostate cancer cells. Values were normalized to Gapdh levels. Experiments were performed in triplicates. Data are representative of 3 independent experiments.

**
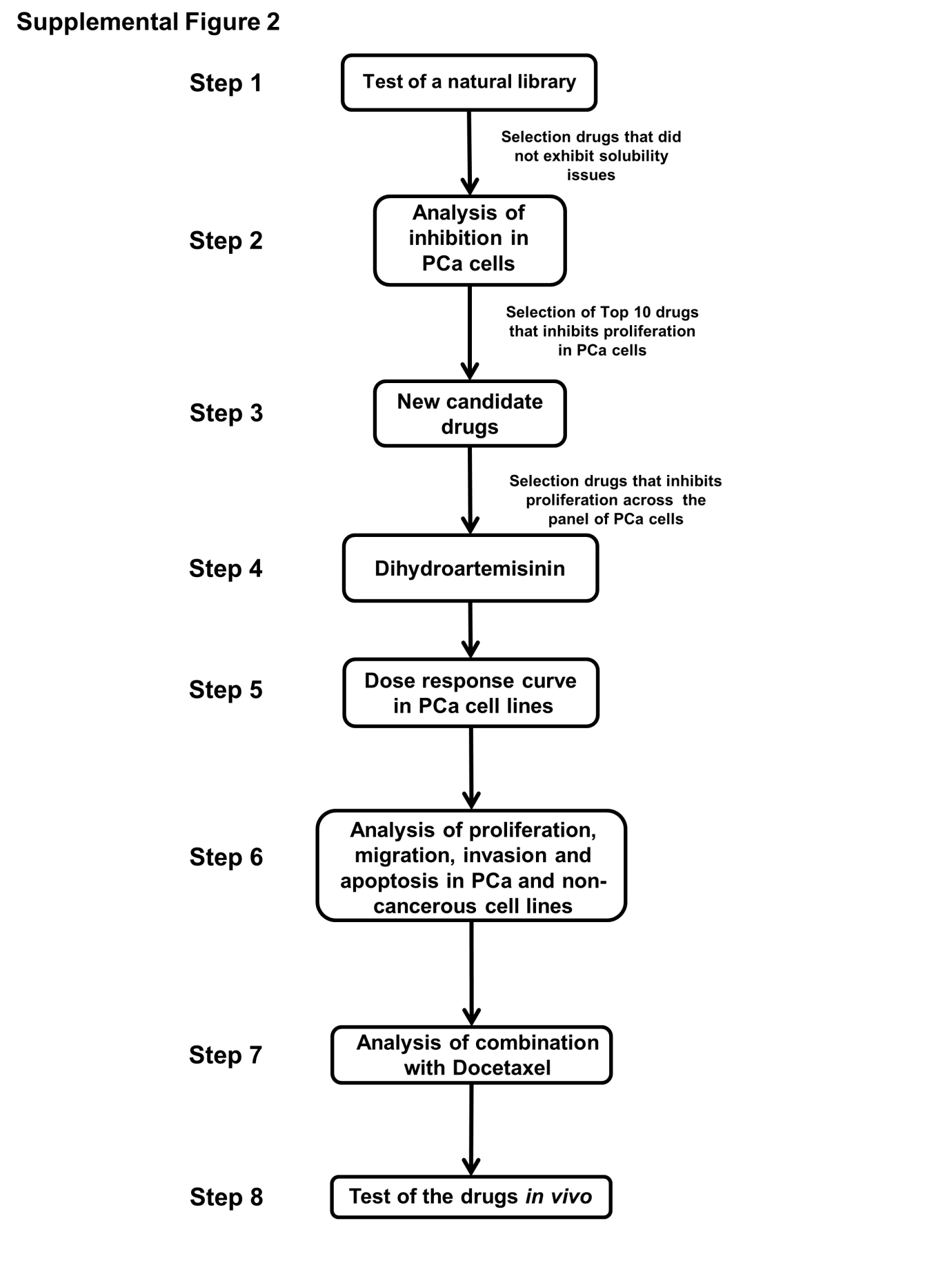
**

Figure S2. Flowchart of study design


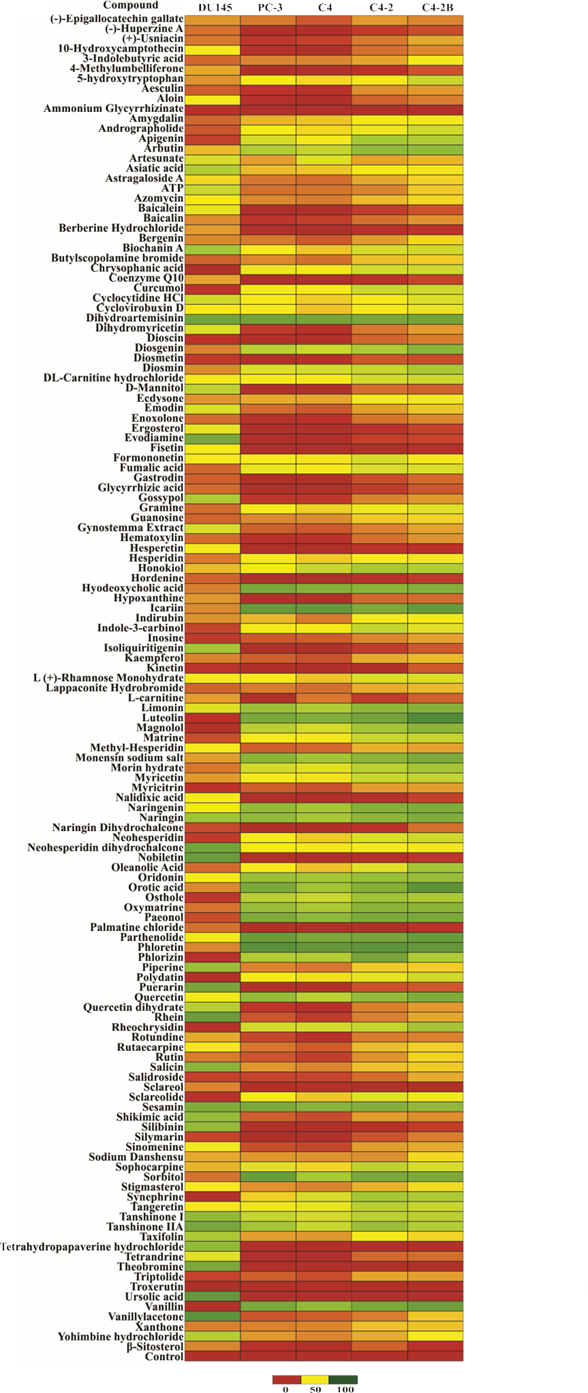


**A**


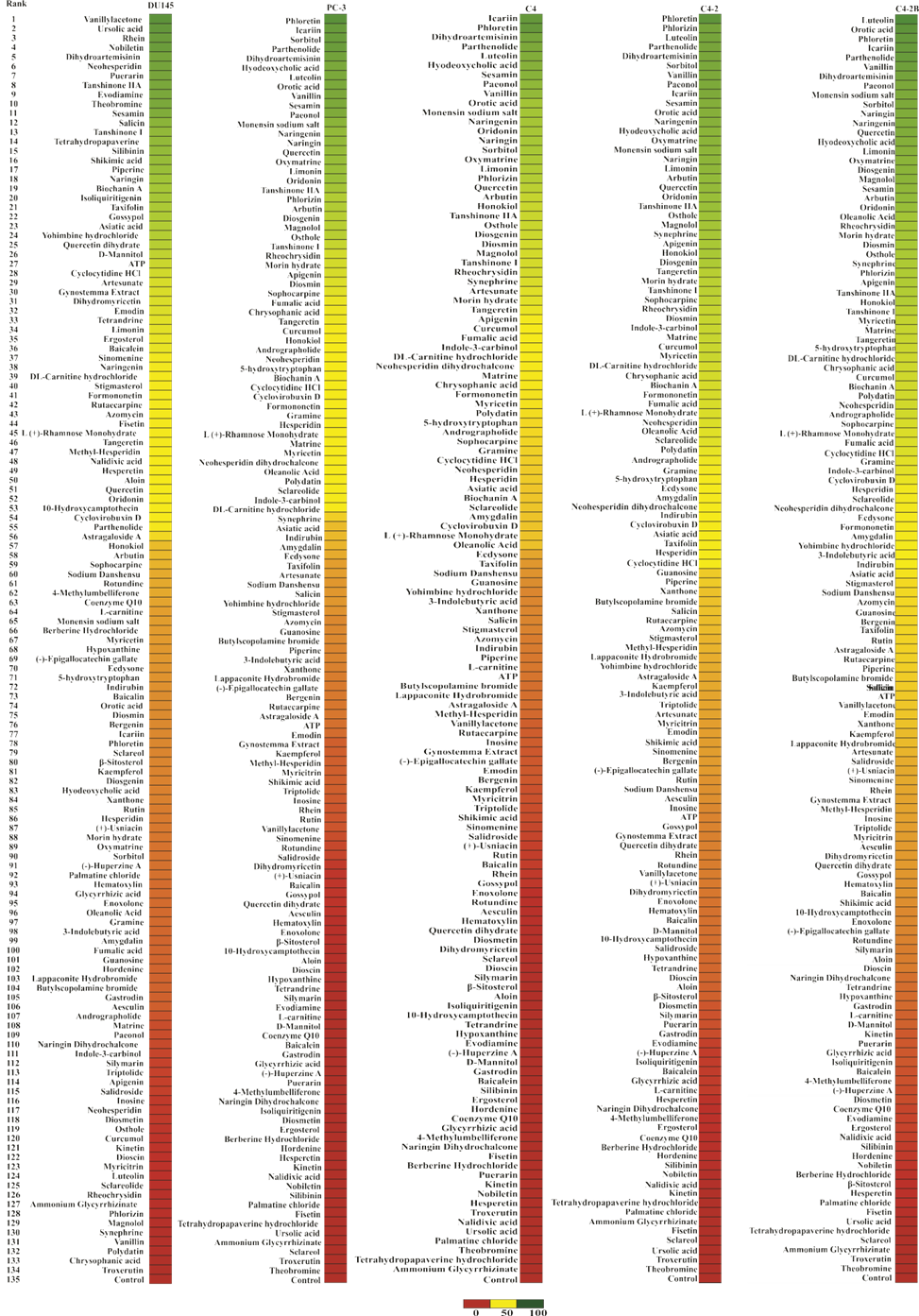


**B**

Figure S3. Analysis of proliferation inhibition from a Natural compound library on PCa cells. (A) Heat map representation of relative proliferation inhibition levels across DU145, PC-3, C4, C4-2 and C4-2B prostate cancer cell lines treated with compounds from a natural compound library. (B) Heat map representation of proliferation inhibition levels in each PCa cell line. Red indicates low inhibition and green indicates relatively high inhibition of proliferation.


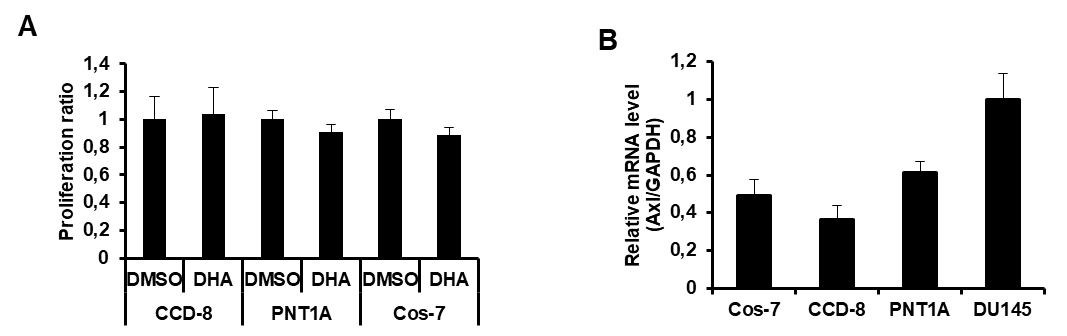


Figure S4. Analysis of non-cancer cell lines (A) Analysis of DHA effects in non-cancer cell lines. Proliferation of CCP, PNT1A, Cos-7 cells treated with 5μM DHA. Data shown are mean ± SD of triplicate independent experiments. Data are representative of 3 independent experiments. (B) qRT-PCR for Axl in non-cancer Values were normalized to Gapdh levels. The experiment was performed in triplicate. Data are representative of 3 independent experiments;


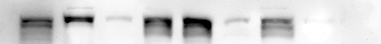

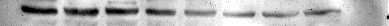


**Axl**

**DU145**

**shGFP shAxl**

**GAPDH**

Figure S5. Axl knodown in PCa cells. Immunoblot analysis of protein extracts obtained from DU145shGFP and DU145shAxl using anti-Axl and anti-GAPDH.


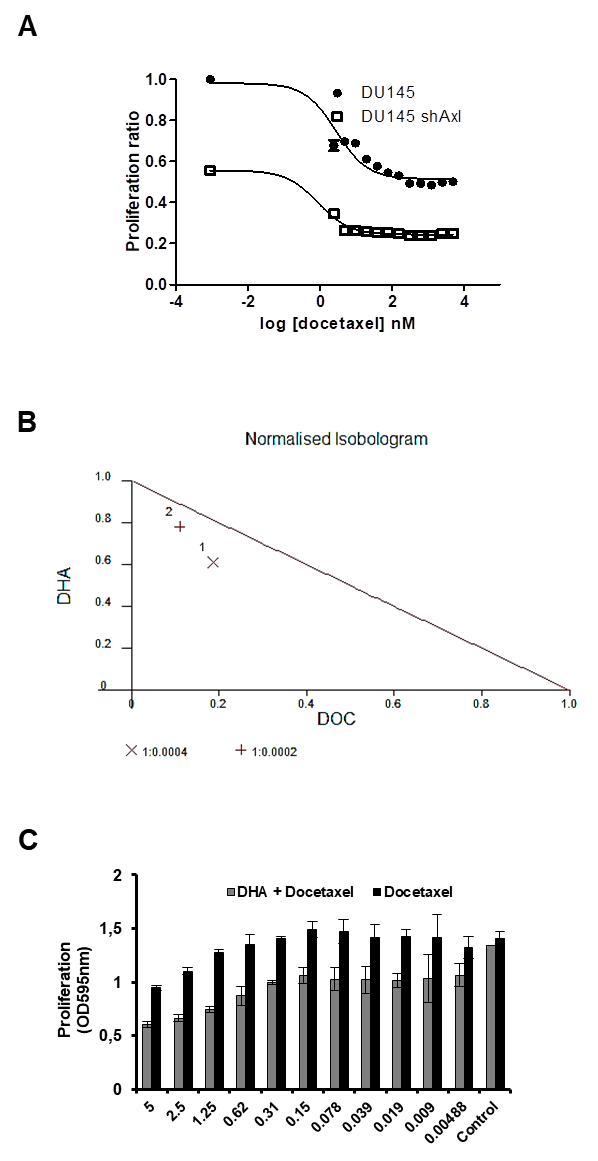


Figure S6. Analysis of docetaxel effects in mCRPCa cell lines. (A) Docetaxel dose-response curve in DU145 and DU145Axl-/-. Proliferation of PCa cells was measured after 24h of treatment with 625, 312.5, 156.3, 78.1, 39.1, 19.5, 9.8, 4.9 and 2.4 nM of docetaxel in order to determine the IC_50_. Data shown are mean ± SD of triplicate independent experiments. (B) Normalized isobologram obtained by software Compusyn. DU145 cells treated with a combination of 2nM of docetaxel and 5μM of DHA and 2nM of docetaxel and 5μM of DHA shows synergistic effect. (C) Docetaxel dose-response curve in DU145 pre-treated with 5μM of DHA. Cells were treated with DHA (5 μM) for 24h and subsequently treated with 5, 2.5, 1.25, 0.62, 0.31, 0.15, 0.078, 0.0390625, 0.019, 0.0097, and 0.0048 µM of docetaxel. Proliferation was measured after 24h of treatment with docetaxel. Data shown are mean ± SD of triplicate independent experiments. Data are representative of 3 independent experiments.


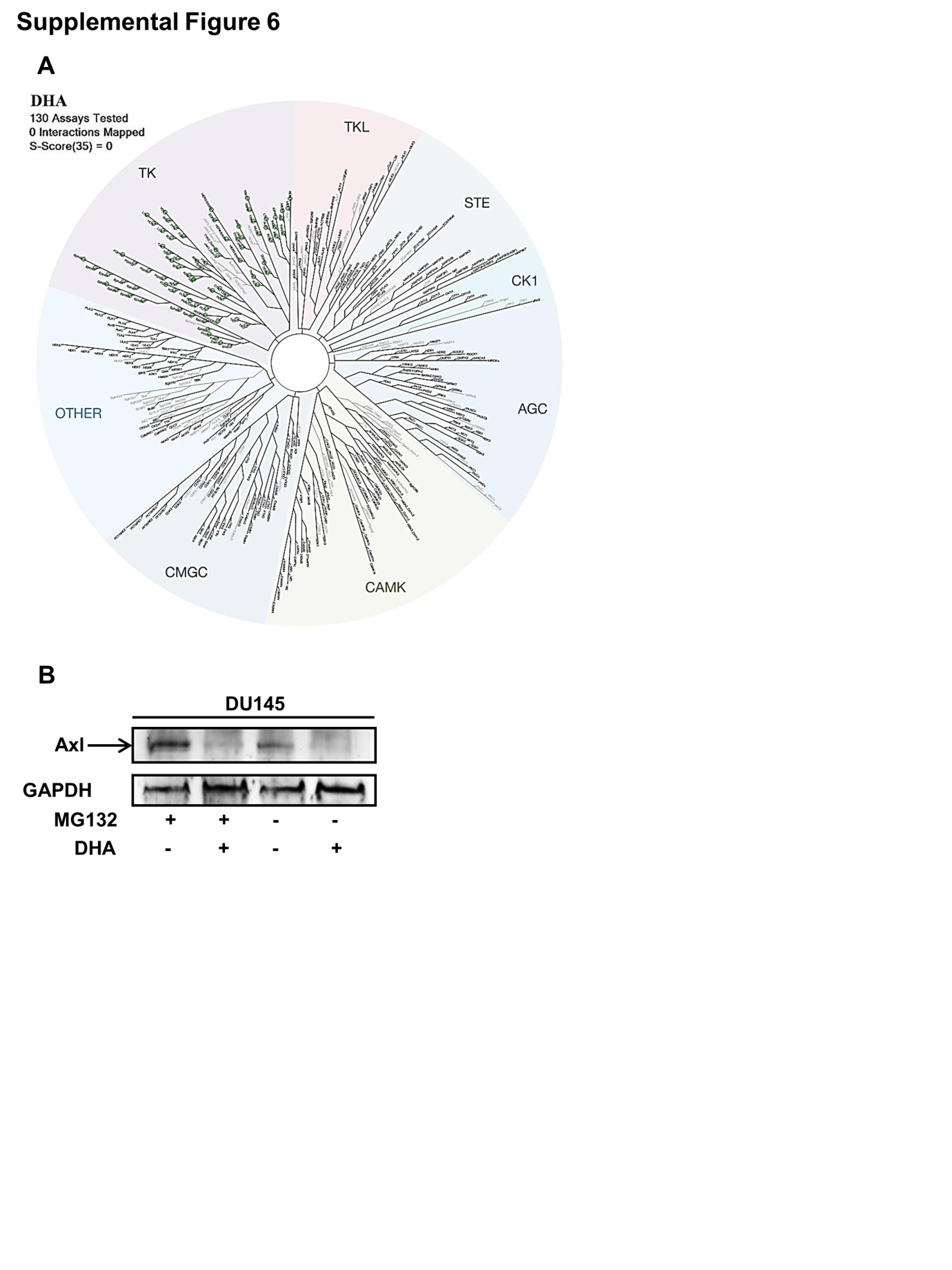


Figure S7. DHA does not inhibit Axl kinase activity in vitro and does not affect the proteasome pathway. (A) DHA-interaction map obtained from analysis using the *scan*TK^℠^ Kinase Assay Panel (DiscoverX) in Du145 dells treated with 5μM of DHA . DHA was found not to bind any kinase tested. (B) Western blot analysis of DU145cells treated with 5μM DHA with or without the proteasome inhibitor MG132 using anti-Axl antibody.


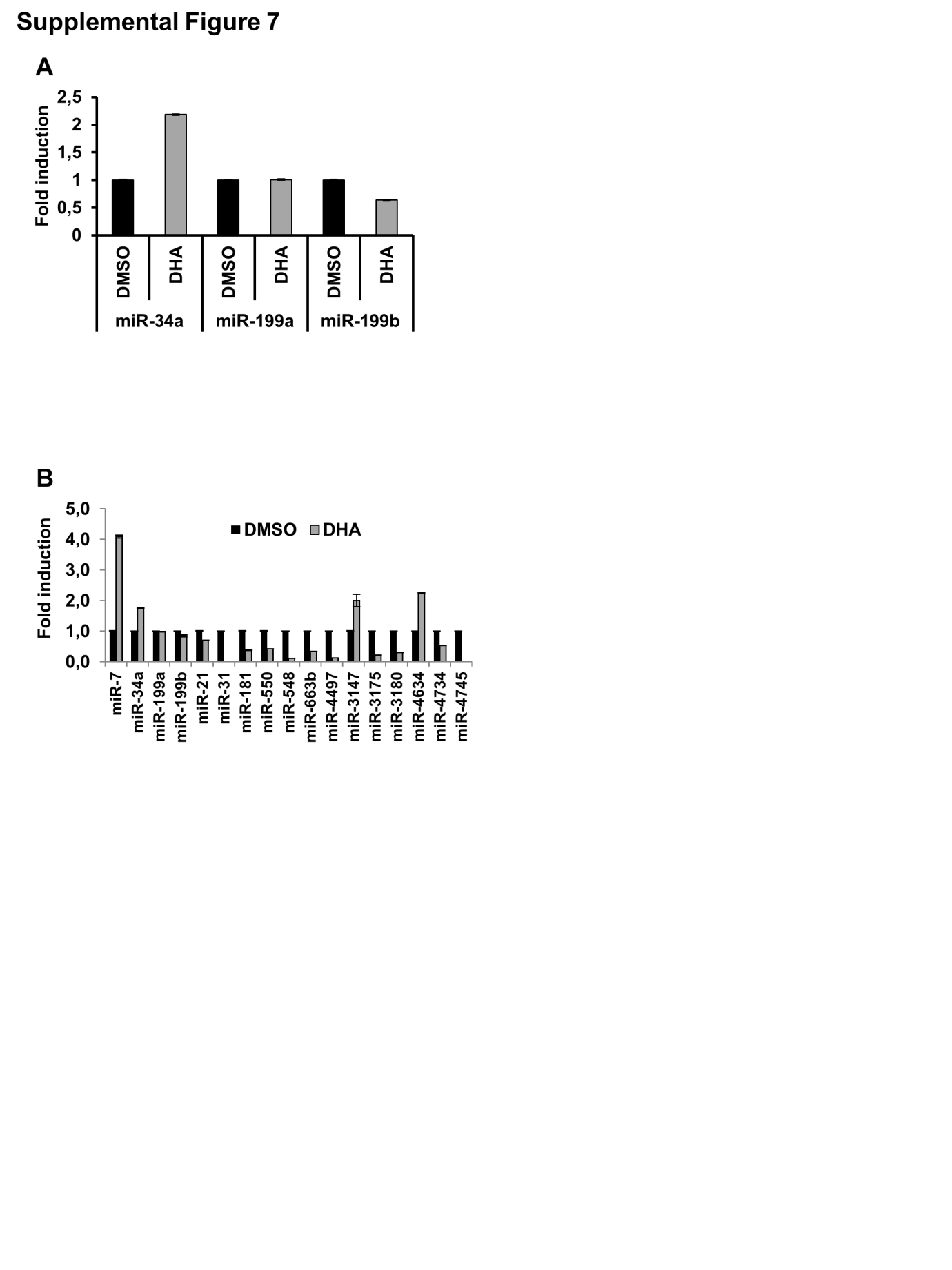


Figure S8. DHA regulates the expression of miRNAs in PCa cell lines. (A) RT-PCR analysis of miR-34a, miR-199a and miR-199b expression in PCa cell lines treated with 5μM DHA. ΔCt values graphed are relative to the endogenous control RNU6B small RNA. Data are representative of 3 independent experiments and all values shown are mean ± SEM from a representative experiment. (B) Validation of miRNAs microarray analysis. PCa cell lines treated with 5μM DHA were analysed by RT-PCR analysis for miR-7, miR-21, miR-31, miR-548, miR-550, miR-3147, miR-3175, miR-3180, miR-4497, miR-4745, miR181a and miR-663b expression. ΔCt values graphed are relative to the endogenous control RNU6B small RNA. Data are representative of 3 independent experiments and all values shown are mean ± SEM from a representative experiment.


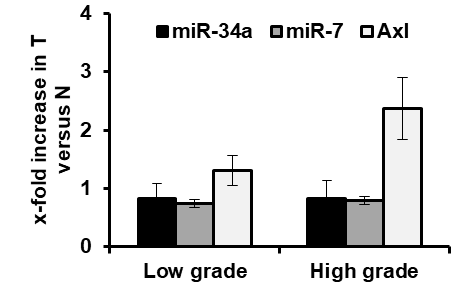


Figure S9..Differential expression mR-34a, miR-7 and Axl in patients tumors of different disease grade. RT-PCR analysis of expression levels of miR-34a, miR-7 and Axl in human PCa samples. Total RNA was collected from human tissue consisting of paired normal and tumor samples and was analyzed for miR-7, miR-34a and Axl expression levels. ΔCt values graphed are relative to the endogenous control RNU6B small RNA (for miR-34a and miR-7) and GAPDH for Axl. Data are shown as the triplicate independent experiments; * p < 0.05, 2-tailed, nonparametric Mann-Whitney test.


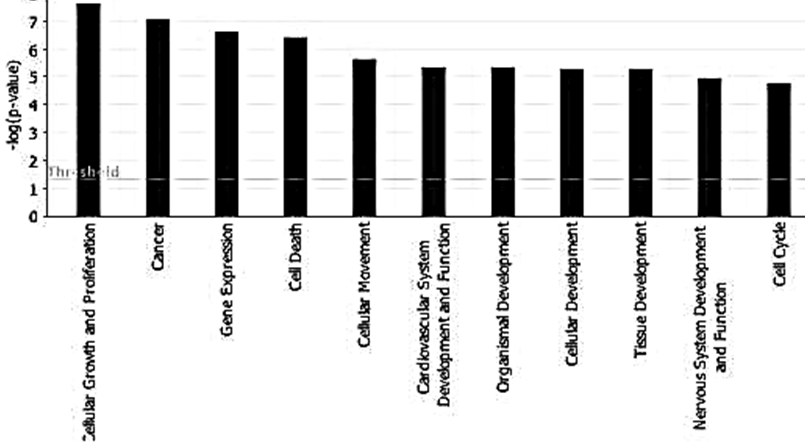


Figure S10. Biological functions activated in response to DHA treatment. The statistical threshold (line without boxes) represents the cut-off for significance on the log scale (y-axis, left side).


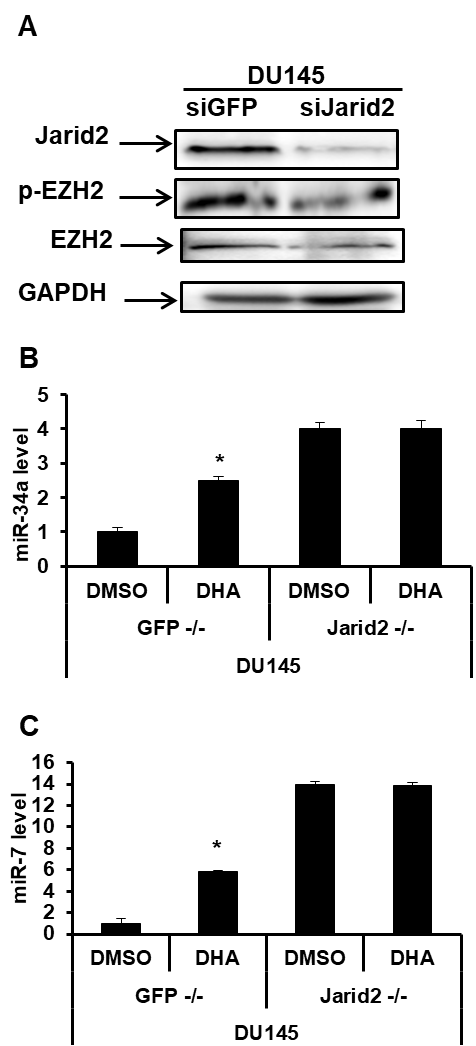


Figure S11. Analysis of inhibition of JARID2 in PCa cell lines. (A) Western blot analysis of JARID2, p-EZH2, EZH2 and Gapdh after transfection of JARID2 or GFP siRNA duplex (50nM) in prostate cancer cell lines. Total RNA and protein were collected from PC-3 cells 24 hours after transfection. (B) and (C) Analysis of miR-34a (B) and miR-7 (C) expression levels in PCa cells treated with 5μM DHA or 0.05% DMSO and transfected with JARID2 or GFP siRNA duplex (50nM). ΔCt values graphed are relative to the endogenous control RNU6B small RNA. Data are representative of 3 independent experiments and all values shown are mean ± SEM from a representative experiment.


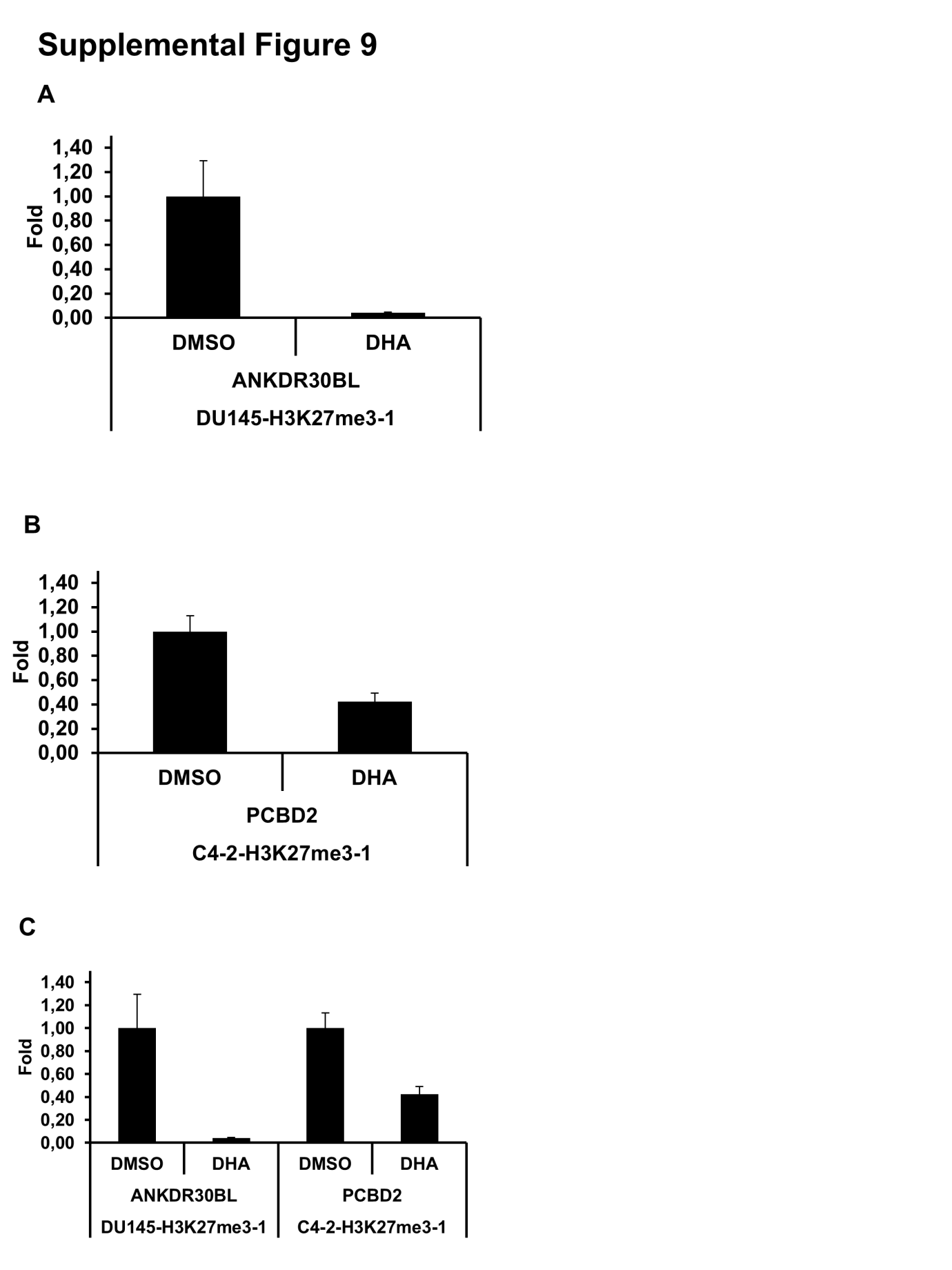


Figure S12. ChIP-qPCR analysis demonstrating the effect of DHA treatment of DU145 and C4-2 on H3K27me3 at specific gene loci. PCa cell lines were treated with 5uM of DHA or DMSO and subjected to ChIP analysis using antibodies against H3K27me3 or control IgG. The signals of H3K27me3 at the indicated genomic locations were normalized to that obtained from IgG ChIP at the same location to calculate their enriched signals.

Table S1 Description of compounds used in the present study

| **Compound** | ***Brief Description*** |
| --- | --- |
| ***(-)-Epigallocatechin gallate*** | (-)-Epigallocatechin gallate is a potent anti-oxidant polyphenol flavonoid isolated from green tea. |
| ***(-)-Huperzine A*** | (-)-Huperzine A is an acetylcholinesterase inhibitor and NMDA receptor antagonist with an IC50 of 126 μM for NMDA-induced current. |
| ***(+)-Usniacin*** | (+)-Usniacin (D-Usnic acid) is a naturally occurring dibenzofuran derivative found in several lichen species. |
| ***10-Hydroxycamptothecin*** | 10-Hydroxycamptothecin (10-HCPT, 10-hydroxy-CPT, OHCPT, NSC107124) is a DNA topoisomerase I inhibitor with potent anti-tumor activity. |
| ***3-Indolebutyric acid*** | 3-Indolebutyric acid (IBA) is a plant hormone in the auxin family and is an ingredient in many commercial plant rooting horticultural products. |
| ***4-Methylumbelliferone*** | 4-Methylumbelliferone is a hyaluronic acid (HA) synthesis inhibitor with an IC50 of 0.4 mM. |
| ***5-hydroxytryptophan*** | 5-Hydroxytryptophan (5-HTP) is decarboxylated to serotonin (5-hydroxytryptamine or 5-HT) by the enzyme aromatic-L-amino-acid decarboxylase with the help of Vitamin B6. |
| ***Aesculin*** | Aesculin (Esculin) is a toxin found in horse chestnuts. |
| ***Aloin*** | Aloin (Barbaloin) is a potent tyrosinase inhibitor with an IC50 of 97 µM for HeLaS3 cells. |
| ***Ammonium Glycyrrhizinate*** | Mono-ammonium glycyrrhizinate (AMGZ) is a unique product derived from liquorice root and has an extremely sweet taste. |
| ***Amygdalin*** | Amygdalin is a glycoside initially isolated from the seeds of the tree Prunus dulcis, also known as bitter almonds. |
| ***Andrographolide*** | Andrographolide is a labdane diterpenoid that is the main bioactive component of the medicinal plant Andrographis paniculata. |
| ***Apigenin*** | Apigenin is a potent CYP2C9 inhibitor. |
| ***Apocynin*** | Apocynin (Acetovanillone) is a selective NADPH-oxidase inhibitor with IC50 of 10 μM. |
| ***Arbutin*** | Arbutin (Uvasol, p-Arbutin) is a tyrosinase inhibitor with an IC50 of 1.09 mM. |
| ***Artesunate*** | Artesunate is a semi-synthetic derivative of artemisinin that treat malaria.. |
| ***Asiatic acid*** | Asiatic acid is the aglycone of asiaticoside isolated from the plant Centella asiatica, commonly used in wound healing. |
| ***Astragaloside A*** | Astragaloside A is a pure small molecular compound isolated from Radix Astragali and is commonly used in the treatment of degenerative bone diseases such as osteoporosis. |
| ***ATP*** | ATP is a disodium salt form of adenosine-triphosphate which is a multifunctional nucleoside triphosphate. |
| ***Azomycin*** | Azomycin (2-Nitroimidazole) is an antimicrobial antibiotic produced by a strain of Nocardia mesenterica. |
| ***Baicalein*** | Baicalein is a CYP2C9 and prolyl endopeptidase inhibitor. |
| ***Baicalin*** | Baicalin is a known prolyl endopeptidase inhibitor and affects the GABA receptors. |
| ***Berberine Hydrochloride*** | Berberine Hydrochloride is a quaternary ammonium salt from the group of isoquinoline alkaloids. |
| ***Bergenin*** | Bergenin (Cuscutin) is trihydroxybenzoic acid glycoside and the C-glycoside of 4-O-methyl gallic acid. |
| ***Bilobalide**** | Bilobalide is a biologically active terpenic trilactone present in Ginkgo biloba. |
| ***Biochanin A*** | Biochanin A (4-Methylgenistein) is an O-methylated isoflavone. It is a natural organic compound in the class of phytochemicals known as flavonoids. |
| ***Butylscopolamine bromide*** | Butylscopolamine bromide, is a peripherally acting antimuscarinic, anticholinergic agent used as an abdominal-specific antispasmodic. |
| ***Caffeic acid**** | Caffeic acid is a hydroxycinnamic acid, a naturally occurring organic compound. |
| ***Chlorogenic acid**** | Chlorogenic acid is a hydroxycinnamic acid and a member of a family of naturally occurring organic compounds. |
| ***Chrysin**** | Chrysin is a naturally occurring flavone chemically extracted from the blue passion flower (Passiflora caerulea). |
| ***Chrysophanic acid*** | Chrysophanic acid (Chrysophanol) is a EGFR/mTOR pathway inhibitor. |
| ***Cinchonidine**** | Cinchonidine is an alkaloid used in asymmetric synthesis in organic chemistry. |
| ***Coenzyme Q10*** | Coenzyme Q10 (ubiquinone, ubidecarenone, coenzyme Q) is a component of the electron transport chain and participates in aerobic cellular respiration. |
| ***Cryptotanshinone**** | Cryptotanshinone is a major tanshinone isolated from Salvia miltiorrhiza that exhibits multiple activities. |
| ***Curcumol*** | Curcumol is a common traditional Chinese medicine with antitumor activities. |
| ***Cyclocytidine HCl*** |  |
| ***Cyclosporin A*** | Cyclosporin A (Cyclosporine A) is an immunosuppressant drug widely used in post-allogeneic organ transplant to reduce the activity of the patient's immune system. |
| ***Cyclovirobuxin D*** | Cyclovirobuxine D (bebuxine; Cyclovirobuxine) is an active compound extracted from Buxus microphylla, which has been used for treating acute myocardial ischemia. |
| ***Cytisine*** | Cytisine is a nicotinic acetylcholine receptor agonist. |
| ***Dihydroartemisinin (DHA)*** | Dihydroartemisinin (DHA) is a semi-synthetic derivative of artemisinin and is isolated from the traditional Chinese herb Artemisia annua. |
| ***Dihydromyricetin*** | Dihydromyricetin (Ampelopsin (flavanol); Ampeloptin) is a natural antioxidant with good prospects. |
| ***Dioscin*** | Dioscin (Collettiside III) is a saponin extracted and isolated from Polygonatum Zanlanscianense Pamp with IC50 of 2.6, 0.8, 7.5, and 4.5 μM for the inhibition of the growth of the MDA-MB-435, H14, HL60, and HeLa cell lines, respectively. |
| ***Diosgenin*** | Diosgenin is a steroid sapogenin and the precursor for the semisynthesis of progesterone which in turn was used in early combined oral contraceptive pills. |
| ***Diosmetin*** | Diosmetin (Luteolin 4-methyl ether) is a bioflavonoid found in spearmint, oregano, and many other plants. |
| ***Diosmin*** | Diosmin is a semisynthetic phlebotropic drug and a member of the flavonoid family. |
| ***DL-Carnitine hydrochloride*** | DL-Carnitine hydrochloride is a quaternary ammonium compound biosynthesized from the amino acids lysine and methionine. |
| ***D-Mannitol*** | D-Mannitol(Osmitrol) is an osmotic diuretic agent and a weak renal vasodilator. |
| ***Ecdysone*** | Ecdysone is a steroidal prohormone of the major insect moulting hormone 20-hydroxyecdysone, which is secreted from the prothoracic glands. |
| ***Emodin*** | Emodin is a purgative resin, 6-methyl-1,3,8-trihydroxyanthraquinone, from rhubarb, the buckthorn and Japanese Knotweed (Fallopia japonica). |
| ***Enoxolone*** | Enoxolone is a pentacyclic triterpenoid derivative of the beta-amyrin type obtained from the hydrolysis of glycyrrhizic acid, which was obtained from the herb liquorice. |
| ***Ergosterol*** | Ergosterol is a sterol and a biological precursor (a provitamin) to vitamin D2. |
| ***Evodiamine*** | Evodiamine (Isoevodiamine) is an alkaloid extract from a plant known as Evodiae Fructus. |
| ***Fisetin*** | Fisetin (Fustel) is a potent sirtuin activating compound (STAC) and an agent that modulates sirtuins. |
| ***Formononetin*** | Formononetin (Formononetol) is a phytoestrogen from the root of Astragalus membranaceus and an O-methylated isoflavone. |
| ***Fumalic acid*** | Fumalic acid (Ferulic acid) is a hydroxycinnamic acid and a type of organic compound found in the Ferula assafoetida L. or Ligusticum chuanxiong. |
| ***Gastrodin*** | Gastrodin (Gastrodine) is a polyphenol. It is the glucoside of 4-hydroxybenzyl alcohol (gastrodigenin). |
| ***Glycyrrhizic acid*** | Glycyrrhizic acid is a widely used anti-inflammatory agent isolated from the liquorice root. |
| ***Gossypol*** | Gossypol is a polyphenolic aldehyde that permeates cells and acts as an inhibitor for several dehydrogenase enzymes. |
| ***Gramine*** | Gramine is a naturally occurring indole alkaloid present in several plant species. |
| ***Guanosine*** |  |
| ***Gynostemma Extract*** | Gynostemma Extract is a saponins extract derived from the Gynostemma pentaphyllum. |
| ***Hematoxylin*** | Hematoxylin (Hydroxybrazilin) is a compound that forms strongly coloured complexes with certain metal ions, notably Fe(III) and Al(III) salts and a kind of stain in histology. |
| ***Hesperetin*** | Hesperetin is a bioflavonoid and, to be more specific, a flavanone. |
| ***Hesperidin*** | Hesperidin is a flavanone glycoside found abundantly in citrus fruits. |
| ***Honokiol*** | Honokiol is a biphenolic compound present in the cones, bark, and leaves of Magnolia grandifloris. |
| ***Hordenine*** | Hordenine (N,N-dimethyl-4-hydroxyphenylethylamine) is a phenylethylamine alkaloid with antibacterial and antibiotic properties. |
| ***Hyodeoxycholic acid*** | Hyodeoxycholic (HDCA) acid is a secondary bile acid, one of the metabolic byproducts of intestinal bacteria. |
| ***Hypoxanthine*** | Hypoxanthine is a naturally occurring purine derivative. |
| ***Icariin*** | Icariin is a flavonol and PDE5 inhibitor with IC50 of 1.0, 0.75, and 1.1 μM for PDE5A1, A2, and A3, respectively. |
| ***Indirubin*** | Indirubin is a potent cyclin-dependent kinases and GSK-3β inhibitor with IC50 of about 75 nM and 0.19 μM. |
| ***Indole-3-carbinol*** | Indole-3-carbinol is produced by the breakdown of the glucosinolate glucobrassicin, which can be found at relatively high levels in cruciferous vegetables. |
| ***Inosine*** | Inosine is a nucleoside that is formed when hypoxanthine is attached to a ribose ring via a β-N9-glycosidic bond. |
| ***Ipriflavone**** | Ipriflavone (7-Isopropoxyisoflavon) is used to inhibit bone resorption. |
| ***Isoliquiritigenin*** | Isoliquiritigenin (GU 17) is a strong nod gene- and glyceollin resistance-inducing flavonoid from soybean root exudate. |
| ***Kaempferol*** | Kaempferol is a natural flavonol, a type of flavonoid, that has been isolated from plant sources. |
| ***Kinetin*** | Kinetin (6-Furfuryladenine) is a kind of cytokinin, a class of plant hormone that promotes cell division. Kinetin is often used in plant tissue culture for inducing formation of callus (in conjunction with auxin) and to regenerate shoot tissues from callus. |
| ***L (+)-Rhamnose Monohydrate*** | L(+)-Rhamnose (Rham) is a naturally-occurring deoxy sugar. |
| ***Lappaconite Hydrobromide*** | Lappaconite Hydrobromide is a kind of alkaloid extracted from Aconitum sinomontanum Nakai and has anti-inflammatory effects. |
| ***L-carnitine*** | L-carnitine (Levocarnitine) is constituent of striated muscle and liver. It is used therapeutically to stimulate gastric and pancreatic secretions and in the treatment of hyperlipoproteinemias. |
| ***Limonin*** | Limonin is also known as limonoate D-ring-lactone and limonoic acid di-delta-lactone. Chemically, it is a member of the class of compounds known as furanolactones. |
| ***Luteolin*** | Luteolin (Luteolol) is a PDE4 inhibitor and a general phosphodiesterase inhibitor, and an Interleukin 6 inhibitor. |
| ***Magnolol*** | Magnolol (2,2'-Bichavicol) is a bioactive compound found in the bark of the Houpu magnolia (Magnolia officinalis). |
| ***Matrine*** | Matrine ((+)-Matrine) is an alkaloid found in plants from the Sophora family. It has a variety of pharmacological effects, including anti-cancer effects, and action as a kappa opioid receptor agonist. |
| ***Methyl-Hesperidin*** | Methyl-Hesperidin is a flavanone glycoside (flavonoid) (C28H34O15) found abundantly in citrus fruits. Its aglycone form is called hesperetin. |
| ***Monensin sodium salt*** | Monensin sodium salt (Coban), isolated from Streptomyces cinnamonensis, is a well-known representative of naturally polyether ionophore antibiotics. |
| ***Morin hydrate*** | Morin hydrate (Aurantica) is a chemical compound. Morin can be used to test for the presence of aluminium or tin in a solution, since it forms characteristically fluorescent coordination complexes with them. |
| ***Myricetin*** | Myricetin (Cannabiscetin) is a naturally occurring flavonol, a flavonoid found in many grapes, berries, fruits, vegetables, herbs, as well as other plants. |
| ***Myricitrin*** | Myricitrin (Myricitrine) is a chemical compound. It can be isolated from the root bark of Myrica cerifera (Bayberry, a small tree native to North America) and in Chrysobalanus icaco. |
| ***Nalidixic acid*** | Nalidixic acid (NegGram) is a synthetic 1,8-naphthyridine antimicrobial agent with a limited bacteriocidal spectrum. It is an inhibitor of the A subunit of bacterial DNA gyrase. |
| ***Naringenin*** | Naringenin is a flavonoid that is considered to have a bioactive effect on human health as antioxidant, free radical scavenger, anti-inflammatory, carbohydrate metabolism promoter, and immune system modulator. |
| ***Naringin*** | Naringin (Naringoside) is a flavanone glycoside. It is a major flavonoid in grapefruit and gives the grapefruit juice its bitter taste. |
| ***Naringin Dihydrochalcone*** | Naringin Dihydrochalcone (Naringin DC) is a new-style sweetening agent and an artificial sweetener derived from naringin. |
| ***Neohesperidin*** | Neohesperidin is an antioxidant agent with an IC50 of 22.31 µg/ml in the 1,1-diphenyl-2-picryldydrazyl (DPPH) radical-scavenging assay. |
| ***Neohesperidin dihydrochalcone*** | Neohesperidin dihydrochalcone (Nhdc), sometimes abbreviated to neohesperidin DC or simply NHDC, is an artificial sweetener derived from citrus. |
| ***Nobiletin*** | Nobiletin (Hexamethoxyflavone) is a chemical compound. It is an O-methylated flavone, a flavonoid isolated from citrus peels, such as tangerine. |
| ***Oleanolic Acid*** | Oleanolic acid (Caryophyllin) is a naturally occurring triterpenoid, widely distributed in food and medicinal plants, related to betulinic acid. It is relatively non-toxic, antitumor, and hepatoprotective, as well as exhibiting antiviral properties. |
| ***Oridonin*** | Oridonin (Isodonol), an entkaurane diterpenoid isolated from Rabdosia rubescens, is an important traditional Chinese herbal remedy. |
| ***Orotic acid*** | Orotic acid (6-Carboxyuracil) is a heterocyclic compound and an acid. |
| ***Osthole*** | Osthole (Osthol) is a type of coumarin. |
| ***Oxymatrine*** | Oxymatrine (Matrine N-oxide) is one of the components of the root of Sophora flavescens. |
| ***Paeonol*** | Paeonol (Peonol) is a major component extracted from Chinese herbs Moutan cortex and Cynanchum paniculatum. |
| ***Palmatine chloride*** | Palmatine hydrochloride (Palmatine chloride (6CI,7CI); Fibrauretin) is a hydrochloride salt of palmatine which is a protoberberine alkaloid. |
| ***Parthenolide*** | Parthenolid e((-)-Parthenolide) is a sesquiterpene lactone which occurs naturally in the plant feverfew (Tanacetum parthenium). |
| ***Dihydronaringenin*** | Phloretin (Dihydronaringenin) is a dihydrochalcone, a type of polyphenol. |
| ***Phlorizin*** | Phlorizin (Phloridzin) is a toxic 2'-glucoside of phloretin. |
| ***Piperine*** | Piperine (1-Piperoylpiperidine) is the alkaloid responsible for the pungency of black pepper and long pepper, along with chavicine (an isomer of piperine). It has also been used in some forms of traditional medicine and as an insecticide. |
| ***Polydatin*** | Polydatin (Piceid), 3,4’,5-trihydroxystilbene-3-β-mono-D-glucoside, the crystal component extracted from the root stem of perennial herbage Polygonum Cuspidatum Sieb.et Zucc. |
| ***Puerarin*** | Puerarin(Kakonein) is a 5-HT2C receptor and benzodiazepine site antagonist. |
| ***Quercetin*** | Quercetin is a PI3K and PKC inhibitor with IC50 of 3.8 μM and 15µg/ml. |
| ***Quercetin dihydrate*** | Quercetin, a flavonol widely distributed in plants. It is an antioxidant, like many other phenolic heterocyclic compounds. |
| ***Rhein*** | Rhein(Monorhein; NSC 38629; Rheic acid; Rheinic acid) is a constituent of rhubarb which was isolated from the fresh rhizome of Rheum coreanum Nakai. |
| ***Rheochrysidin*** | Rheochrysidin (Physcion; physcione; parietin) is one of the major components of Da-Cheng-Qi decoction used in the treatment of inflammation. |
| ***Rotundine*** | Rotundine (L-tetrahydropalmatine, L-THP) is a selective dopamine D1 receptor antagonist with IC50 of 166 nM. |
| ***Rutaecarpine*** | Rutaecarpine is an indolopyridoquinazolinone alkaloid isolated from Evodia rutaecarpa and related herbs. |
| ***Rutin*** | Rutin, a flavonol glycoside found in many plants, including buckwheat; tobacco; forsythia; hydrangea; viola, etc. |
| ***Salicin*** | Salicin (Salicoside, Salicine) is an alcoholic β-glycoside that contains D-glucose. Salicin is an anti-inflammatory agent that is produced from willow bark. |
| ***Salidroside*** | Salidroside (Rhodioloside; Rhodosin) is a glucoside compound with an IC50 of 4.99 ± 0.23 μg/mL for the proliferation of SACC-2 cells. |
| ***Sclareol*** | Sclareol is a fragrant chemical compound found in clary sage (Salvia sclarea), from which it derives its name. It is classified as a bicyclic diterpene alcohol. |
| ***Sclareolide*** | Sclareolide (Norambreinolide) is a sesquiterpene lactone natural product derived from various plant sources including Salvia sclarea, Salvia yosgadensis, and cigar tobacco. |
| ***Sesamin*** | Sesamin (Fagarol) is a lignin isolated from the bark of Fagara plants and from sesame oil. |
| ***Shikimic acid*** | Shikimic acid (Shikimate), more commonly known in its anionic form shikimate, is an important biochemical intermediate in plants and microorganisms. |
| ***Silibinin*** | Silibinin (Silybin) is the major active constituent of silymarin. |
| ***Silymarin*** | Silymarin (Silybin B), a strong novel free radical scavenger, is used for treatment of patients with acute brain infarction. |
| ***Sinomenine*** | Sinomenine (Cucoline) is traditionally used in herbal medicine, as a treatment for rheumatism and arthritis. |
| ***Sodium Danshensu*** | Sodium Danshensu is a mono sodium of danshensu which is a compound isolated from Salvia miltiorrhiza Bge. |
| ***Sophocarpine*** | Sophocarpine is a human ether-à-go-go-related gene (HERG) inhibitor with an IC(50) of about 0.2 mM. |
| ***Sorbitol*** | Sorbitol (Glucitol) is a sugar alcohol and a sugar substitute. |
| ***Stigmasterol*** | Stigmasterol (Stigmasterin) is used as a precursor in the manufacture of synthetic progesterone. |
| ***Synephrine*** | Synephrine (Oxedrine) is a drug commonly used for weight loss. |
| ***Tangeretin*** | Tangeretin (Tangeritin) is a citrus flavonoid. |
| ***Tanshinone I*** | Tanshinone I is isolated from Danshen. |
| ***Tanshinone IIA*** | Tanshinone IIA (Tanshinone B) is the most abundant diterpene quinone in Danshen, Salviae miltiorrhizae Radix, a widely prescribed traditional herbal medicine that is used to treat cardiovascular and inflammatory diseases. |
| ***Dihydroquercetin*** | Taxifolin(Dihydroquercetin) is a flavanonol, a type of flavonoid. |
| ***Tetrahydropapaverine hydrochloride*** | Tetrahydropapaverine, one of the TIQs and an analogue of salsolinol and tetrahydropapaveroline, has been reported to have neurotoxic effects on dopamine neurons. |
| ***Tetrandrine*** | Tetrandrine (Fanchinine; Hanfangchin A) is a calcium channel blocker with an IC50 of 38.23± 25.77 μM. |
| ***Theobromine*** | Theobromine (3,7-Dimethylxanthine) is a xanthine alkaloid that is used as a bronchodilator and as a vasodilator. |
| ***Triptolide*** |  |
| ***Troxerutin*** | Troxerutin (Trihydroxyethylrutin) is a flavonol, a type of flavonoid. |
| ***Ursolic acid*** | Ursolic acid (Malol) is a pentacyclic triterpene acid, used in cosmetics. |
| ***Vanillin*** | Vanillin is a phenolic aldehyde used as a flavouring agent in foods, beverages, and pharmaceuticals. |
| ***Vanillylacetone*** | Vanillylacetone is similar in chemical structure to other flavour chemicals such as vanillin and eugenol. It is used as a flavour additive in spice oils and in perfumery to introduce spicy aromas. |
| ***Xanthone*** | Xanthone is an organic compound. It can be prepared by the heating of phenyl salicylate. |
| ***Yohimbine hydrochloride*** | Yohimbine (Antagonil) has been used as a mydriatic and in the treatment of impotence. It is also alleged to be an aphrodisiac. |
| ***β-Sitosterol*** | β-Sitosterol is one of several phytosterols (plant sterols) with chemical structures similar to that of cholesterol. |

Table S2 – Summary of the analysis of a natural library of compounds in Prostate cancer cell lines

|  | | | Number of compounds | | |
| --- | --- | --- | --- | --- | --- |
|  | **DU145** | **PC-3** | **C4** | **C4-2** | **C4-2B** |
| Insoluble/total tested* | 10/144 | 10/144 | 10/144 | 10/144 | 10/144 |

* The compounds exhibiting solubility problems in the conditions tested were Apocynin, Bilobalide, Caffeic acid, Chlorogenic acid, Chrysin, Cinchonidine, Cryptotanshinone, Cyclosporin, Cytisine and Ipriflavone.

Table S3 – Summary of top 15 drugs able to inhibit proliferation of PCa cells (DU145, PC-3, C4, C4-2 and C4-2B). Proliferation was measured 24h post treatment with 10µM of each compound. Percentage of inhibition is related to cells treated with DMSO as control.

| **Rank** | **Compound** (% of inhibition) | | | | |
| --- | --- | --- | --- | --- | --- |
|  | DU145 | PC-3 | C4 | C4-2 | C4-2B |
| **1** | Vanillylacetone | Dihydronaringenin | Icariin | Dihydronaringenin | Luteolin |
|  | (82) | (82) | (80) | (81) | (84) |
| **2** | Ursolic acid | Icariin | Dihydronaringenin | Phlorizin | Orotic acid |
|  | (80) | (79) | (80) | (76) | (81) |
| **3** | Monorhein | Sorbitol | DHA | Luteolin | Dihydronaringenin |
|  | (79) | (79) | (76) | (76) | (80) |
| **4** | Hexamethoxyflavone | Parthenolide | Parthenolide | Parthenolid | Icariin |
|  | (78) | (78) | (75) | (76) | (79) |
| **5** | DHA | DHA | Luteolin | DHA | Parthenolide |
|  | (76) | (77) | (74) | (75) | (79) |
| **6** | Neohesperidin dihydrochalcone | Hyodeoxycholic acid | Hyodeoxycholic acid | Sorbitol | Vanillin |
|  | (76) | (75) | (72) | (75) | (78) |
| **7** | Puerarin | Luteolin | Sesamin | Vanillin | DHA |
|  | (76) | (75) | (72) | (75) | (76) |
| **8** | Tanshinone IIA | Orotic acid | Paeonol | Paeonol | Paeonol |
|  | (75) | (75) | (70) | (74) | (75) |
| **9** | Evodiamine | Vanillin | Vanillin | Icariin | Monensin sodium salt |
|  | (75) | (75) | (70) | (74) | (75) |
| **10** | Theobromine | Sesamin | Orotic acid | Sesamin | Sorbitol |
|  | (75) | (74) | (67) | (73) | (74) |

Table S4 – Clinical information from each patient sample used in the present study

| sample | Gleason | Stage | Grade |
| --- | --- | --- | --- |
| 1 | 3+3 | T3aNx | low grade |
| 2 | 3+4 | T2aNx | low grade 3 dominate |
| 3 | 3+4 | T2c Nx | low grade 3 dominate |
| 4 | 3+4 | T2Nx | low grade 3 dominate |
| 5 | 3+4 | T3bNx | low grade 3 dominate |
| 6 | 3+4 | T2a Nx | low grade 3 dominate |
| 7 | 4+3 | T3a Nx | high grade 4 dominant |
| 8 | 3+3 | T2c Nx | low grade |
| 9 | 4+5 | T2aN0(0/3) | high grade |
| 10 | 4+3 | T2c Nx | high grade 4 dominant |
| 11 | 4+4 | T2aNx | high grade |
| 12 | 4+4 | T2a Nx | high grade |
| 13 | 5+4 | T3bN0(0/14) | high grade |
| 14 | 5+4 | T2aNx | high grade |
| 15 | 4+4 | T3bN1(1/8) | high grade |
| 16 | 4+4 | T2c Nx | high grade |
